# Development of a Decision Model to Estimate the Outcomes of Treatment Sequences in Advanced Melanoma

**DOI:** 10.1177/0272989X251319338

**Published:** 2025-02-22

**Authors:** Saskia de Groot, Hedwig M. Blommestein, Brenda Leeneman, Carin A. Uyl-de Groot, John B. A. G. Haanen, Michel W. J. M. Wouters, Maureen J. B. Aarts, Franchette W. P. J. van den Berkmortel, Willeke A. M. Blokx, Marye J. Boers-Sonderen, Alfons J. M. van den Eertwegh, Jan Willem B. de Groot, Geke A. P. Hospers, Ellen Kapiteijn, Olivier J. van Not, Astrid A. M. van der Veldt, Karijn P. M. Suijkerbuijk, Pieter H. M. van Baal

**Affiliations:** Institute for Medical Technology Assessment (iMTA), Erasmus University Rotterdam, The Netherlands; Erasmus School of Health Policy and Management, Erasmus University Rotterdam, The Netherlands; Erasmus School of Health Policy and Management, Erasmus University Rotterdam, The Netherlands; Institute for Medical Technology Assessment (iMTA), Erasmus University Rotterdam, The Netherlands; Erasmus School of Health Policy and Management, Erasmus University Rotterdam, The Netherlands; Institute for Medical Technology Assessment (iMTA), Erasmus University Rotterdam, The Netherlands; Erasmus School of Health Policy and Management, Erasmus University Rotterdam, The Netherlands; Division of Medical Oncology, Netherlands Cancer Institute, Amsterdam, The Netherlands; Scientific Bureau, Dutch Institute for Clinical Auditing, Leiden, The Netherlands; Department of Surgical Oncology, Netherlands Cancer Institute, Antoni van Leeuwenhoek, Amsterdam, The Netherlands; Department of Biomedical Data Sciences, Leiden University Medical Center, Leiden, The Netherlands; Department of Medical Oncology, GROW School for Oncology and Developmental Biology, Maastricht University Medical Center+, Maastricht, The Netherlands; Department of Medical Oncology, Zuyderland Medical Center Sittard, Sittard-Geleen, The Netherlands; Department of Pathology, Division of Laboratories, Pharmacy and Biomedical Genetics, University Medical Center Utrecht, Utrecht, The Netherlands; Department of Medical Oncology, Radboud University Medical Center, Nijmegen, The Netherlands; Department of Medical Oncology, Amsterdam UMC, VU University Medical Center, Cancer Center Amsterdam, Amsterdam, The Netherlands; Isala Oncology Center, Isala, Zwolle, The Netherlands; Department of Medical Oncology, University Medical Center Groningen, University of Groningen, Groningen, The Netherlands; Department of Medical Oncology, Leiden University Medical Center, Leiden, The Netherlands; Scientific Bureau, Dutch Institute for Clinical Auditing, Leiden, The Netherlands; Department of Medical Oncology, University Medical Center Utrecht, Utrecht, The Netherlands; Department of Medical Oncology and Radiology & Nuclear Medicine, Erasmus Medical Center, Rotterdam, The Netherlands; Department of Medical Oncology, University Medical Center Utrecht, Utrecht, The Netherlands; Erasmus School of Health Policy and Management, Erasmus University Rotterdam, The Netherlands

**Keywords:** Advanced melanoma, decision model, treatment sequences, semi-Markov, immunotherapies, BRAF plus MEK inhibitors

## Abstract

**Background:**

A decision model for patients with advanced melanoma to estimate outcomes of a wide range of treatment sequences is lacking.

**Objectives:**

To develop a decision model for advanced melanoma to estimate outcomes of treatment sequences in clinical practice with the aim of supporting decision making. The article focuses on methodology and long-term health benefits.

**Methods:**

A semi-Markov model with a lifetime horizon was developed. Transitions describing disease progression, time to next treatment, and mortality were estimated from real-world data (RWD) as a function of time since starting treatment or disease progression and patient characteristics. Transitions were estimated separately for melanoma with and without a BRAF mutation and for patients with favorable and intermediate prognostic factors. All transitions can be adjusted using relative effectiveness of treatments derived from a network meta-analysis of randomized controlled trials (RCTs). The duration of treatment effect can be adjusted to obtain outcomes under different assumptions.

**Results:**

The model distinguishes 3 lines of systemic treatment for melanoma with a BRAF mutation and 2 lines of systemic treatment for melanoma without a BRAF mutation. Life expectancy ranged from 7.8 to 12.0 years in patients with favorable prognostic factors and from 5.1 to 8.7 years in patients with intermediate prognostic factors when treated with sequences consisting of targeted therapies and immunotherapies. Scenario analyses illustrate how estimates of life expectancy depend on the duration of treatment effect.

**Conclusion:**

The model is flexible because it can accommodate different treatments and treatment sequences, and the duration of treatment effects and the transitions influenced by treatment can be adjusted. We show how using RWD and data from RCTs can harness advantages of both data sources, guiding the development of future decision models.

**Highlights:**

- The model is flexible because it can accommodate different treatments and treatment sequences, and the duration of treatment effects as well as the transitions that are influenced by treatment can be adjusted.
- The long-term health benefits of treatment sequences depend on the place of different therapies within a treatment sequence.
- Assumptions about the duration of relative treatment effects influence the estimates of long-term health benefits.
- We show how the use of real-world data and data from randomized controlled trials harness the advantages of both data sources, guiding the development of future decision models.

## Introduction

Decision models are developed to support health care decision making by integrating different data sources, extrapolating data over a longer time horizon, and estimating uncertainty. For a long time, health care decisions had to be made between a limited number of alternative treatment options, often guided by evidence from a randomized controlled trial (RCT) to inform a decision model. However, as the number of treatment options has increased, models comparing only 2 alternatives have become inadequate as these may lead to suboptimal decision making and health losses.^1,2^ When multiple treatment options are available, clinicians and policy makers need guidance on the added value of a new treatment compared with all relevant alternatives as well as guidance on the optimal positioning of this new treatment within a sequence of treatments.^3^ This highlights the need for models that compare multiple treatments as well as sequences of treatments. Because the choice and effectiveness of treatments often depend on patient and disease characteristics, it is important to incorporate these in such models.^4^

Decision models provide a convenient structure to integrate multiple data sources. While RCTs are most likely to provide unbiased estimates of treatment effects,^5,6^ they have notable limitations when they are used for other purposes than demonstrating efficacy. First, results from RCTs typically present the average effect for selected patients treated under optimal conditions, which may not accurately represent outcomes of patients treated in clinical practice. Second, despite publications of extended follow-up (≥5 years) for some RCTs, the follow-up is usually too short to fully inform decision models. Real-world data can address these limitations by providing insights about a broader, unselected patient population treated in everyday settings. Increasingly, real-world data are used alongside trial data to improve decision making^7^ and to verify long-term extrapolation of trial data.^8^ By integrating trial data and real-world data in decision models, these models ensure that estimates of treatment effects are grounded in robust evidence while also delivering the comprehensive insights decision makers need, such as lifetime outcomes of patients in clinical practices.

Advanced melanoma (i.e., unresectable stage III and stage IV) is one of the indications in oncology where patient and disease characteristics (e.g., Eastern Cooperative Oncology Group [ECOG] performance status, BRAF mutation status, lactate dehydrogenase [LDH] level, and presence of [symptomatic] brain metastases) inform decisions about treatment and are associated with survival.^9^ Since 40% of the advanced melanoma patients are not eligible for RCTs given their patient and disease characteristics, complementing data from RCTs with real-world data is particularly relevant for this indication.^10^ Furthermore, advanced melanoma has faced rapid advances in the treatment landscape in the past decade with the introduction of immunotherapies and targeted therapies.^11,12^ Ipilimumab, pembrolizumab, and nivolumab (plus ipilimumab) received market authorization for patients with advanced melanoma. For patients with advanced melanoma and a BRAF mutation, additional treatments targeting this mutation have been approved, such as vemurafenib (plus cobimetinib), dabrafenib (plus trametinib), and encorafenib plus binimetinib.

Melanoma patients do not only have more treatment options nowadays,^13,14^ but they are also receiving more successive lines of treatment than 10 years ago. A limited number of decisions models are available that were developed with the aim of evaluating treatments for advanced melanoma.^15^ Most of these did not model treatment sequences,^16–21^ and problems were identified with the level of detail in reporting outcomes.^20,21^ The models for treatment sequences^22–27^ did not include all currently relevant treatments (e.g., nivolumab plus ipilimumab and dabrafenib plus trametinib) or allow for distinguishing between types of anti-PD1 and targeted therapies in the sequences.^25,27^ Furthermore, the method of modeling effects of (subsequent) treatments was critiqued^22^ as well as the level of details reported on the methods.^23,28^ Finally, these sequence models relied either entirely on RCT^23,24,26^ or real-world data,^25,27^ hampering both the generalizability and opportunity to correct relative treatment effectiveness for possible imbalances due to unmeasured confounders.

The objective of this study was to develop a decision model for advanced melanoma to estimate the long-term health benefits of treatment sequences in clinical practice, to support clinicians and policy makers in their decision- aking. In this article, we present the structure of the model, its methodology, the long-term health benefits, and the impact of different assumptions.

## Methods

### Target Population and Subgroups

Since patient and disease characteristics (i.e., prognostic factors) of advanced melanoma patients determine the available treatment options, we distinguished, based on clinical expert opinion supported by real-world evidence,^9,29,30^ between patients with favorable and intermediate prognostic factors. Patients with favorable prognostic factors have a normal serum LDH level, an ECOG performance status of 0 or 1, and no brain metastases. Patients with intermediate prognostic factors have an LDH elevation, although less than or equal to twice the upper limit of normal, an ECOG performance status of 0 or 1, and no or asymptomatic brain metastases. Also, patients with a normal LDH level, an ECOG performance status of 0 or 1, but asymptomatic brain metastases were classified as patients with intermediate prognostic factors. Patients with poor prognostic factors (i.e., LDH elevation larger than twice the upper limit of normal; an ECOG performance status of 2, 3, or 4; or symptomatic brain metastases) were excluded, because these patients were also mostly excluded from phase III RCTs, and therefore, estimates of treatment effects within these patients were unavailable. We further divided patients into 2 groups: patients with a BRAF mutation (i.e., all BRAF mutations [BRAF V600 and BRAF nonV600]) versus patients without a BRAF mutation (i.e., BRAF wild-type melanoma).

### Model Structure

**Figure 1.**
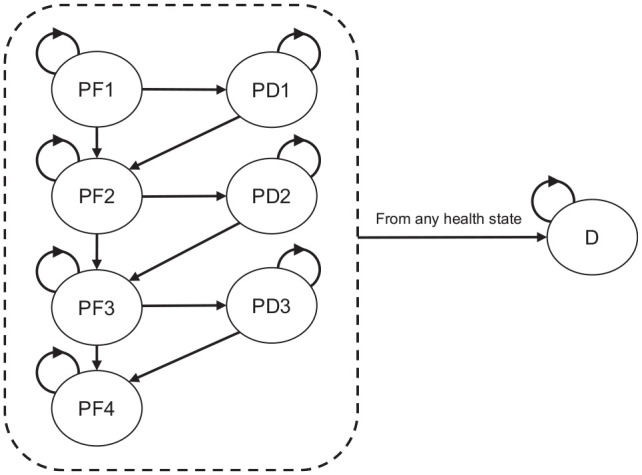
Model structure advanced melanoma with a BRAF mutation. PF1, progression free during or after treatment line 1; PD1, progressive disease after treatment line 1; PF2, progression free during or after treatment line 2; PD2, progressive disease after treatment line 2; PF3, progression free during or after treatment line 3; PD3, progressive disease after treatment line 3; PF4, progression free during and progressive disease after subsequent therapies; D, death. Death is an absorbing state; patients can transition to this state from anywhere in the model. A similar model structure has been used for advanced melanoma without a BRAF mutation but with 2 instead of 3 lines of systemic treatment (i.e., without PD3 and PF4).

To estimate the long-term health benefits of treatment sequences, a Markov model was developed. Markov models are able to capture the continuous risk of events that are associated with changes in health states, and they are commonly used to evaluate treatments for patients with melanoma^15^ and many other diseases. Health states in the model were defined based on events relevant to the disease course: progression free, progression of disease, and death. Figure 1 shows the structure of the model for advanced melanoma with a BRAF mutation. The model distinguishes a maximum of 3 lines of systemic treatment including 8 health states and 16 possible transitions between health states. The model does not explicitly consider the treatment effect of fourth and subsequent lines because real-world data showed that only 10% of the patients received more than 3 lines of treatment. For advanced melanoma without a BRAF mutation, the model distinguishes 2 lines of systemic treatment and includes 6 health states and 11 possible transitions between health states.

Because the transitions in the model from progressive disease depend on the time spent in this state, duration dependency was incorporated by tunnel states, which changed our model to a semi-Markov model. Furthermore, a clock-reset approach was required to model the progression of disease, subsequent treatment, and death as a function of the time since the start of a treatment line (i.e., the start of a second or third treatment line). The semi-Markov model was run separately for patients with favorable and intermediate prognostic factors and for patients with and without a BRAF mutation. The time horizon of the model was lifetime.

The model allows all transitions between health states to be a function of treatment. This means all transitions to other health states can be adjusted for the effectiveness of treatment (e.g., more effective treatments will lead to lower risks of a next event such as progression of disease or death). The model further has the flexibility to adapt the duration of treatment effect.

### Estimating Transitions from Real-World Data

Since we aimed to estimate the outcomes of sequences of treatments for patients in clinical practice, we used data from RCTs and real-world data. Real-world data were obtained from the nationwide Dutch Melanoma Treatment Registry (DMTR) to inform parameters describing prognostic factors as well as parameters describing disease progression, treatment status, and mortality (data cutoff: April 4, 2021).^31^ The DMTR has been recording nationwide data from patients treated for advanced melanoma in all 14 designated melanoma centers in the Netherlands since 2012. We included all adult patients with advanced cutaneous melanoma who were treated with systemic treatment between 2012 and 2020 and who were not treated within a trial (patients in early access and/or compassionate use programs were included). A total of 2,549 patients met the eligibility criteria: 1,500 patients with a BRAF mutation and 1,049 patients without a BRAF mutation (see Supplementary Figure 1.1 for reasons of exclusion). Patient and disease characteristics are shown in Supplementary Table 1.1. Most of the patients had favorable prognostic characteristics (63%). The treatments these patients received are presented in Supplementary Table 2.1. Of the patients, 58% and 28% with a BRAF mutation and 31% and 7% of patients without a BRAF mutation received a second and third treatment line, respectively.

We estimated times to events (i.e., progression of disease, a new treatment, or death) from data from the DMTR to inform the transitions between health states. The DMTR included dates of starting treatment, progression of disease per line of treatment, and death. When progression of disease was registered on the same day that a patient received a new treatment, it was assumed that progression of disease occurred 1 d before the start of a new treatment. Similarly, when progression of disease was registered on the same day that a patient died, it was assumed that progression of disease occurred 1 d before death. Patients who did not experience the event of interest during follow-up (e.g., progression of disease) were censored at either one of the next events (e.g., a new treatment or death) or at the end of follow-up when no next event occurred (i.e., the date of the last registered visit to the melanoma center).

All transitions were specified by a variety of parametric survival models, including generalized gamma, generalized F, Weibull, gamma, exponential, log-logistic, log-normal, and Gompertz. Patient and disease characteristics (i.e., age, sex, ECOG performance status, LDH level, and brain metastases at baseline) were included as covariates. The fit of different models was assessed systematically by evaluating the Akaike information criterion and Bayesian information criterion. In addition, visual inspection informed how well the parametric survival models fitted the data and was used to explore the extrapolation over time. The final fit of the outcome of the Markov model to the observed OS was decisive in choosing between different parametric models. Details about the fit of different models can be found in Supplementary Table 3.1 to 3.12 and Supplementary Figure 3.1 to 3.8. Supplementary Table 4.1 shows which parametric models were used for all the transitions in the model, separately for melanoma with a BRAF mutation and melanoma without a BRAF mutation and for patients with favorable and intermediate prognostic factors.

In compliance with Dutch regulations, the DMTR was approved by a medical ethical committee and was not subject to the Medical Research Involving Human Subjects Act.

### Modeling Treatment Effectiveness

Hazard functions describing the transitions to move from one state to another in the model estimated from the DMTR were subsequently adjusted to make predictions for a scenario in which patients receive a certain treatment using relative treatment effects estimated from a network meta-analysis (NMA)^11^ in the following manner:

(1) $$h_{j,i}(t)\frac{HR_{j}}{\sum_{j=1}^{n}\;p_{j,i}HR_{j}}\times h_{i}(t)$$

where $h_{j,i}(t)$ denotes the transition intensity at time *t* for a patient with characteristics *i* who receives treatment *j*, $p_{j,i}$ the proportion of patients with characteristics *i* who receive treatment *j*, $HR_{j}$ indicating the relative treatment effect expressed in a hazard ratio (HR) where *j* is an index for treatments and *j* *=* *1* is the reference treatment (dacarbazine) and $h_{i}(t)$ denotes the transition intensity at time *t* for a patient with characteristics *i*. $p_{j,i}$ and $h_{i}(t)$ are estimated from the DMTR, and $HR_{j}$ were derived from an NMA combining data from 17 trials for 19 treatment options.^11^

Given that the hazard functions were estimated from data where patients receive different treatments, these hazard functions can be interpreted as a weighted average of hazard functions of patients receiving different treatments. Consequently, we cannot directly multiply the estimated hazard function with a HR from the NMA as this would overestimate the effectiveness of treatments. Therefore, we divided $HR_{j}$ with the mean HR of the population from which the transitions are estimated (denoted $\sum_{j=1}^{n}\;p_{j,i}HR_{j}$ ). Note that for observable factors that could influence disease progression/mortality (and thus transitions in the model) irrespectively of type of treatment received (i.e., age, sex, ECOG performance status, LDH level, and brain metastases), we included covariates (captured in subscript *i*). We did not include covariates for treatments because treatments in daily practice are not randomly assigned. Such covariates are therefore likely to correlate strongly with unobservable factors (e.g., patient preferences and doctor knowledge).

### Model Calculation

First, we computed the values of the hazard functions over time, using monthly cycles, for all transitions and all treatment sequences, given the parametric survival models we selected based on real-world data. When the extrapolated rates of death from the parametric survival models became lower than age-specific mortality rates from the general Dutch population, the mortality rates from Statistics Netherlands were used. Transition matrices were created for every time point, separately for patients starting a first, second, and/or third treatment line. Then, Markov traces were obtained providing the estimated number of patients in each of the health states over time for all treatment lines and all different treatments. Finally, these Markov traces were reduced to one trace, by weighing the Markov traces by the proportion of patients who received a subsequent treatment for all cycles.

The mean age and proportion of men and women that we assumed for the predictions were based on Dutch-specific real-world data (Supplementary Table 1.1), separately for the different subgroups. For example, we estimated outcomes for patients with a BRAF mutation and favorable prognostic factors being 60 years old and assuming 56% being men.

Data from the DMTR were prepared in STATA statistical analysis software (StataCorp 2019, Stata Statistical Software: Release 16; StataCorp LLC, College Station, TX, USA). The statistical analyses and model programming were completed using RStudio version 4.3.2 (RStudio Team 2020, RStudio: Integrated Development for R; RStudio, PBC, Boston, MA, USA).

### Base-Case Analyses

Outcomes of current treatment (which did not require an adjustment of the HRs with equation 1) as well as outcomes of 21 treatment sequences for melanoma with a BRAF mutation and 8 treatment sequences for melanoma without a BRAF mutation were estimated, separately for patients with favorable and intermediate prognostic factors. Treatments and treatment sequences were selected if they seemed relevant from a clinical point of view, which means that treatments that were no longer prescribed were not taken into account in the evaluation. Supplementary Table 5.1 and 5.2 provide an overview of the modeled treatment sequences.

Table 1 provides an overview of the base-case settings and assumptions. In the base case, we applied the HRs for progression-free survival (PFS) from the NMA^11^ and assumed that due to a change in treatment, the hazard rates for the transition from progression free to progressive disease change (from PF to PD), that is, when a patient receives a more effective treatment, his or her probability to progress becomes lower. The HRs for targeted therapies (i.e., BRAF and MEK inhibitors), based on the NMA, are most favorable, but clinical experts expect this effect to last for a shorter time. We therefore assumed that the relative effectiveness regarding the PFS (i.e., the HR as obtained from the NMA) of immunotherapies (i.e., anti-CTLA-4 and anti-PD-1 antibodies) lasts for 2 years, whereas the relative effectiveness regarding the PFS of targeted therapies lasts for 6 mo. In other words, the transition probabilities were adjusted for 2 and 0.5 year, respectively, which allowed for crossing of PF curves. This assumption with respect to the duration of the relative effectiveness was based on the outcomes of the DREAMseq trial^32^ and expert consultation. The DREAMseq trial showed that during the first 6 mo, nivolumab plus ipilimumab was less effective in terms of PFS compared with dabrafenib plus trametinib. After that, however, the PFS curves cross, and from 8 mo onward, nivolumab plus ipilimumab resulted in more durable and ongoing responses compared with dabrafenib plus trametinib.

**Table 1:** Base-Case Settings and Assumptions of the Cost-Effectiveness Model and Scenario Analyses

Base-case assumptions	
Relative effectiveness	Results from an NMA of 17 phase III trials: • HR PFS from NMA (figure 4^11^) applies to transition PF to PD *Duration* of treatment effect: • Chemotherapy: 2 years • Immunotherapies: 2 years • Targeted therapies: 0.5 years HR OS from NMA (figure 5^11^) applies to transitions PF to D and PD to D *Duration* of treatment effect: • All therapies: until start of a new treatment line The relative effect of treatment in treatment-naïve patients is representative for the effect of a treatment in a sequence, regardless of treatment line. Hence, the relative treatment effect is assumed to be independent of the type and number of previous therapies.
Treatment duration	Immunotherapies: maximum of 2 years Targeted therapies: • until progression/death or start new treatment line • stopped after 5 years for patients who remain progression free
Time horizon	Lifetime
Scenario analyses	
Scenario 1	Duration of relative effectiveness of targeted therapy regarding PFS holds 0.25 year (instead of 0.5 year)
Scenario 2	Duration of relative effectiveness of targeted therapy regarding PFS holds 1 year (instead of 0.5 year)
Scenario 3	Duration of relative effectiveness of immunotherapy regarding OS holds beyond line of therapy (instead of within line of therapy)
Scenario 4	Time horizon of 10 years

D, death; DMTR, Dutch Melanoma Treatment Registry; HR, hazard ratio; NMA, network meta-analysis; OS, overall survival; PD, progressive disease; PF, progression free; PFS, progression-free survival.

In the base case, we also applied the HRs for OS from the NMA^11^ and assumed that due to a change in treatment, the hazard rates for the transition from progression free and progressive disease to death change (from PF to D and from PD to D). For all therapies, it was assumed that the relative effectiveness regarding OS lasts until patients start a new treatment line (until patients enter PF2 or PF3).

Furthermore, we assumed that the relative treatment effect (obtained from the NMA in treatment-naive patients [main network]) is representative for the relative treatment effect of second- and third-line therapies, meaning that the relative treatment effects were assumed to be independent of (the type of) previous treatment. We also assumed that the relative treatment effects were independent of prognostic factors. To include encorafenib plus binimetinib, we assumed that the effectiveness (i.e., HR PFS and HR OS) of encorafenib plus binimetinib is like the effectiveness of dabrafenib plus trametinib.

### Sensitivity and Scenario Analyses

To account for the uncertainty of the parameter estimates, probabilistic sensitivity analyses (PSAs) were conducted, taking into account the uncertainty regarding the relative effectiveness of the treatments and the initial hazard functions. The uncertainty regarding the relative effectiveness of the treatments was accounted for by drawing HRs from the 95% credible intervals from the NMA. Uncertainty in the initial hazard functions, as obtained from the DMTR, was handled using the Cholesky decomposition of the variance-covariance matrices. We have used 1,000 iterations for each PSA.

Scenario analyses were conducted for all treatment sequences to demonstrate the impact of alternative assumptions regarding the impact of treatments on PFS and OS on mean life expectancy (Table 1).

### Role of the Funder

This work was supported by an unrestricted grant from the Erasmus University Medical Centre. The funder had no role in the design and conduct of the study; collection, management, analysis, and interpretation of the data; preparation, review, or approval of the manuscript; or decision to submit the manuscript for publication.

## Results

### Model Outcomes and Validation with Real-World Data

If patients were treated in the model following treatment frequencies as observed in clinical practice, the median OS of patients with a BRAF mutation with favorable or intermediate prognostic factors was estimated to be 33 and 18 mo, respectively. For comparison, data from the DMTR showed a median OS of 32 mo (95% confidence interval [CI]: 27–40) and 16 mo (95% CI: 15–19). With the model, the mean OS of patients with a BRAF mutation with favorable and intermediate prognostic factors was estimated to be 9.4 and 6.1 years, respectively.

If patients were treated in the model following treatment frequencies as observed in clinical practice, the median OS of patients without a BRAF mutation with favorable and intermediate prognostic factors was 30 and 16 mo, respectively. In these subgroups, real-world data showed a median OS of 33 mo (95% CI: 25–44 mo) and 15 mo (95% CI: 12–21 mo), respectively. With the model, the mean OS of patients without a BRAF mutation with favorable and intermediate prognostic factors was estimated to be 7.1 and 5.6 years, respectively.

### Effectiveness of Treatment Sequences

Table 2 shows the life expectancy of patients with a BRAF mutation and favorable or intermediate prognostic factors and the impact of different treatment sequences. The table also shows the mean time spent in each health state. The life expectancy ranged from 8.6 to 12.0 years in patients with favorable prognostic factors and from 5.1 to 7.7 years in patients with intermediate prognostic factors when they are treated with treatment sequences consisting of targeted therapies and immunotherapies. These treatment sequences outweigh the life expectancy based on chemotherapy only (i.e., 3.6 and 1.7 years in patients with favorable and intermediate prognostic factors, respectively).

**Table 2:** Life Expectancy in Years and Mean Time Spent in Different Health States of Patients with a BRAF Mutation Stratified by Prognostic Factors: Results of the Base Case

First-Line Treatment	Mix (Observed in Clinical Practice)^a^	Chemotherapy	Dabrafenib plus Trametinib			Vemurafenib plus Cobimetinib			Encorafenib plus Binimetinib		
Second-Line Treatment	Mix (Observed in Clinical Practice)^a^	Chemotherapy	Nivolumab	Pembrolizumab	Nivolumab plus Ipilimumab	Nivolumab	Pembrolizumab	Ipilimumab plus Nivolumab	Nivolumab	Pembrolizumab	Nivolumab plus Ipilimumab
Third-Line Treatment	Mix (Observed in Clinical Practice)^a^	Chemotherapy	Real-World Treatment Mix^b^	Real-World Treatment Mix^b^	Real-World Treatment Mix^b^	Real-World Treatment Mix^b^	Real-World Treatment Mix^b^	Real-World Treatment Mix^b^	Real-World Treatment Mix^b^	Real-World Treatment Mix^b^	Real-World Treatment Mix^b^
Patients with favorable prognostic factors											
Life expectancy in years (95% CI)	9.4 (8.2–10.6)	3.6 (2.9–4.3)	10.7 (9–12.4)	10.4 (8.6–12.2)	11.5 (9.6–13.4)	10.6 (8.9–12.3)	10.3 (8.5–12.2)	11.4 (9.5–13.3)	10.7 (9–12.4)	10.4 (8.6–12.2)	11.5 (9.6–13.4)
Mean time in PF1 in years (95% CI)	3.5 (2.7–4.3)	0.9 (0.7–1.1)	4.5 (3.3–5.6)	4.5 (3.3–5.6)	4.5 (3.3–5.6)	4.4 (3.2–5.5)	4.4 (3.2–5.5)	4.4 (3.2–5.5)	4.5 (3.3–5.6)	4.5 (3.3–5.6)	4.5 (3.3–5.6)
Mean time in PD1 in years (95% CI)	1.2 (0.8–1.6)	0.9 (0.5–1.3)	1.2 (0.7–1.7)	1.2 (0.7–1.7)	1.2 (0.7–1.7)	1.2 (0.7–1.6)	1.2 (0.7–1.6)	1.2 (0.7–1.6)	1.2 (0.7–1.7)	1.2 (0.7–1.7)	1.2 (0.7–1.7)
Mean time in PF2 in years (95% CI)	1.8 (1.2–2.3)	0.3 (0.3–0.4)	1.9 (1.1–2.7)	1.7 (0.7–2.7)	2.6 (1.5–3.8)	1.9 (1.1–2.7)	1.7 (0.7–2.7)	2.6 (1.5–3.8)	1.9 (1.1–2.7)	1.7 (0.7–2.7)	2.6 (1.5–3.8)
Mean time in PD2 in years (95% CI)	0.5 (0.2–0.8)	0.3 (0.1–0.4)	0.7 (0.3–1.1)	0.7 (0.2–1.1)	0.8 (0.3–1.2)	0.7 (0.3–1.1)	0.7 (0.2–1.1)	0.8 (0.3–1.2)	0.7 (0.3–1.1)	0.7 (0.2–1.1)	0.8 (0.3–1.2)
Mean time in PF3 in years (95% CI)	0.8 (0.4–1.3)	0.1 (0.1–0.1)	0.8 (0.4–1.3)	0.8 (0.4–1.3)	0.8 (0.4–1.3)	0.8 (0.4–1.3)	0.8 (0.4–1.3)	0.8 (0.4–1.3)	0.8 (0.4–1.3)	0.8 (0.4–1.3)	0.8 (0.4–1.3)
Mean time in PD3 in years (95% CI)	0.3 (0.1–0.5)	0.1 (0–0.2)	0.3 (0.1–0.6)	0.3 (0.1–0.6)	0.3 (0.1–0.6)	0.3 (0.1–0.6)	0.3 (0.1–0.6)	0.3 (0.1–0.6)	0.3 (0.1–0.6)	0.3 (0.1–0.6)	0.3 (0.1–0.6)
Mean time in PF4 in years (95% CI)	1.3 (0.7–1.9)	1 (0.6–1.5)	1.3 (0.7–1.9)	1.3 (0.7–1.9)	1.3 (0.7–1.9)	1.3 (0.7–1.9)	1.3 (0.7–1.9)	1.3 (0.7–1.9)	1.3 (0.7–1.9)	1.3 (0.7–1.9)	1.3 (0.7–1.9)
2-y survival	63%	26%	70%	69%	72%	69%	68%	72%	70%	69%	72%
5-y survival	36%	11%	41%	40%	44%	41%	40%	44%	41%	40%	44%
Patients with intermediate prognostic factors											
Life expectancy in years (95% CI)	6.1 (4.8–7.3)	1.7 (1.2–2.3)	6.8 (5.1–8.5)	6.7 (5–8.4)	7.4 (5.6–9.2)	6.7 (5.1–8.3)	6.6 (4.9–8.2)	7.3 (5.5–9.1)	6.8 (5.1–8.5)	6.7 (5–8.4)	7.4 (5.6–9.2)
Mean time in PF1 in years (95% CI)	2.7 (1.8–3.6)	0.4 (0.3–0.5)	3.4 (2.1–4.6)	3.4 (2.1–4.6)	3.4 (2.1–4.6)	3.3 (2.1–4.6)	3.3 (2.1–4.6)	3.3 (2.1–4.6)	3.4 (2.1–4.6)	3.4 (2.1–4.6)	3.4 (2.1–4.6)
Mean time in PD1 in years (95% CI)	1.3 (0.9–1.8)	0.8 (0.4–1.3)	1.3 (0.7–1.8)	1.3 (0.7–1.8)	1.3 (0.7–1.8)	1.2 (0.7–1.8)	1.2 (0.7–1.8)	1.2 (0.7–1.8)	1.3 (0.7–1.8)	1.3 (0.7–1.8)	1.3 (0.7–1.8)
Mean time in PF2 in years (95% CI)	0.9 (0.5–1.3)	0.1 (0.1–0.1)	0.7 (0.3–1.2)	0.7 (0.1–1.2)	1.2 (0.4–2)	0.7 (0.3–1.2)	0.7 (0.1–1.2)	1.2 (0.4–2)	0.7 (0.3–1.2)	0.7 (0.1–1.2)	1.2 (0.4–2)
Mean time in PD2 in years (95% CI)	0.3 (0.1–0.6)	0.1 (0–0.2)	0.5 (0.1–0.9)	0.4 (0–0.9)	0.6 (0.1–1.1)	0.5 (0.1–0.9)	0.4 (0–0.9)	0.6 (0.1–1.1)	0.5 (0.1–0.9)	0.4 (0–0.9)	0.6 (0.1–1.1)
Mean time in PF3 in years (95% CI)	0.4 (0.1–0.7)	0 (0–0)	0.5 (0.1–0.8)	0.5 (0.1–0.8)	0.5 (0.1–0.8)	0.5 (0.1–0.8)	0.4 (0.1–0.8)	0.5 (0.1–0.8)	0.5 (0.1–0.8)	0.5 (0.1–0.8)	0.5 (0.1–0.8)
Mean time in PD3 in years (95% CI)	0 (0–0.1)	0 (0–0)	0 (0–0.1)	0 (0–0.1)	0 (0–0.1)	0 (0–0.1)	0 (0–0.1)	0 (0–0.1)	0 (0–0.1)	0 (0–0.1)	0 (0–0.1)
Mean time in PF4 in years (95% CI)	0.4 (0–0.8)	0.3 (0–0.5)	0.4 (0–0.8)	0.4 (0–0.8)	0.4 (0–0.9)	0.4 (0–0.8)	0.4 (0–0.8)	0.4 (0–0.9)	0.4 (0–0.8)	0.4 (0–0.8)	0.4 (0–0.9)
2-y survival	43%	10%	48%	47%	51%	47%	46%	50%	48%	47%	51%
5-y survival	25%	6%	27%	27%	29%	27%	26%	29%	27%	27%	29%
First-Line Treatment	Nivolumab				Pembrolizumab				Nivolumab plus Ipilimumab		
Second-Line Treatment	Ipilimumab	Dabrafenib plus Trametinib	Vemurafenib plus Cobimetinib	Encorafenib plus Binimetinib	Ipilimumab	Dabrafenib plus Trametinib	Vemurafenib plus Cobimetinib	Encorafenib plus Binimetinib	Dabrafenib plus Trametinib	Vemurafenib plus Cobimetinib	Encorafenib plus Binimetinib
Third-Line Treatment	Real-World Treatment Mix^b^	Real-World Treatment Mix^b^	Real-World Treatment Mix^b^	Real-World Treatment Mix^b^	Real-World Treatment Mix^b^	Real-World Treatment Mix^b^	Real-World Treatment Mix^b^	Real-World Treatment Mix^b^	Real-World Treatment Mix^b^	Real-World Treatment Mix^b^	Real-World Treatment Mix^b^
Patients with favorable prognostic factors											
Life expectancy in years (95% CI)	9 (7.2–10.8)	10.8 (9.2–12.5)	10.7 (9.1–12.4)	10.8 (9.2–12.5)	8.6 (6.4–10.8)	10.4 (8.5–12.4)	10.3 (8.3–12.4)	10.4 (8.5–12.4)	12 (10–14)	11.9 (9.9–13.9)	12 (10–14)
Mean time in PF1 in years (95% CI)	4 (2.6–5.3)	4 (2.6–5.3)	4 (2.6–5.3)	4 (2.6–5.3)	3.6 (1.8–5.3)	3.6 (1.8–5.3)	3.6 (1.8–5.3)	3.6 (1.8–5.3)	5.2 (3.3–7.1)	5.2 (3.3–7.1)	5.2 (3.3–7.1)
Mean time in PD1 in years (95% CI)	1.4 (0.9–2)	1.4 (0.9–2)	1.4 (0.9–2)	1.4 (0.9–2)	1.4 (0.8–2)	1.4 (0.8–2)	1.4 (0.8–2)	1.4 (0.8–2)	1.5 (0.9–2)	1.5 (0.9–2)	1.5 (0.9–2)
Mean time in PF2 in years (95% CI)	0.6 (0.2–1)	2.5 (1.6–3.3)	2.4 (1.6–3.2)	2.5 (1.6–3.3)	0.6 (0.3–1)	2.5 (1.6–3.3)	2.4 (1.6–3.2)	2.5 (1.6–3.3)	2.4 (1.6–3.2)	2.3 (1.5–3.1)	2.4 (1.6–3.2)
Mean time in PD2 in years (95% CI)	0.5 (0.1–0.9)	0.5 (0.2–0.9)	0.5 (0.2–0.9)	0.5 (0.2–0.9)	0.5 (0.1–0.9)	0.5 (0.2–0.9)	0.5 (0.2–0.9)	0.5 (0.2–0.9)	0.5 (0.2–0.9)	0.5 (0.2–0.8)	0.5 (0.2–0.9)
Mean time in PF3 in years (95% CI)	0.9 (0.4–1.3)	0.8 (0.4–1.3)	0.8 (0.4–1.3)	0.8 (0.4–1.3)	0.9 (0.4–1.3)	0.8 (0.4–1.3)	0.8 (0.4–1.3)	0.8 (0.4–1.3)	0.8 (0.4–1.2)	0.8 (0.4–1.2)	0.8 (0.4–1.2)
Mean time in PD3 in years (95% CI)	0.3 (0.1–0.6)	0.3 (0.1–0.5)	0.3 (0.1–0.5)	0.3 (0.1–0.5)	0.3 (0.1–0.6)	0.3 (0.1–0.6)	0.3 (0.1–0.5)	0.3 (0.1–0.6)	0.3 (0.1–0.5)	0.3 (0.1–0.5)	0.3 (0.1–0.5)
Mean time in PF4 in years (95% CI)	1.3 (0.7–1.9)	1.3 (0.7–1.9)	1.3 (0.7–1.9)	1.3 (0.7–1.9)	1.3 (0.7–2)	1.3 (0.7–1.9)	1.3 (0.7–1.9)	1.3 (0.7–1.9)	1.3 (0.7–1.8)	1.2 (0.7–1.8)	1.3 (0.7–1.8)
2-y survival	59%	69%	69%	69%	57%	68%	67%	68%	73%	73%	73%
5-y survival	33%	41%	41%	41%	31%	40%	39%	40%	45%	45%	45%
Patients with intermediate prognostic factors											
Life expectancy in years (95% CI)	5.4 (3.8–6.9)	6.5 (4.9–8.1)	6.5 (4.9–8)	6.5 (4.9–8.1)	5.1 (3.3–6.9)	6.2 (4.3–8)	6.1 (4.3–8)	6.2 (4.3–8)	7.7 (5.7–9.6)	7.6 (5.7–9.6)	7.7 (5.7–9.6)
Mean time in PF1 in years (95% CI)	2.3 (1.2–3.4)	2.3 (1.2–3.4)	2.3 (1.2–3.4)	2.3 (1.2–3.4)	2.1 (0.7–3.4)	2.1 (0.7–3.4)	2.1 (0.7–3.4)	2.1 (0.7–3.4)	3.3 (1.6–5.1)	3.3 (1.6–5.1)	3.3 (1.6–5.1)
Mean time in PD1 in years (95% CI)	1.7 (1.1–2.3)	1.7 (1.1–2.3)	1.7 (1.1–2.3)	1.7 (1.1–2.3)	1.6 (0.9–2.4)	1.6 (0.9–2.4)	1.6 (0.9–2.4)	1.6 (0.9–2.4)	1.8 (1.2–2.4)	1.8 (1.2–2.4)	1.8 (1.2–2.4)
Mean time in PF2 in years (95% CI)	0.2 (0.1–0.3)	1.3 (0.6–1.9)	1.2 (0.6–1.9)	1.3 (0.6–1.9)	0.2 (0.1–0.3)	1.3 (0.6–1.9)	1.2 (0.6–1.9)	1.3 (0.6–1.9)	1.3 (0.6–2)	1.3 (0.6–1.9)	1.3 (0.6–2)
Mean time in PD2 in years (95% CI)	0.2 (0–0.5)	0.3 (0–0.7)	0.3 (0–0.6)	0.3 (0–0.7)	0.2 (0–0.5)	0.3 (0–0.7)	0.3 (0–0.6)	0.3 (0–0.7)	0.3 (0–0.7)	0.3 (0–0.7)	0.3 (0–0.7)
Mean time in PF3 in years (95% CI)	0.4 (0.1–0.8)	0.4 (0.1–0.8)	0.4 (0.1–0.8)	0.4 (0.1–0.8)	0.4 (0.1–0.8)	0.4 (0.1–0.8)	0.4 (0.1–0.8)	0.4 (0.1–0.8)	0.5 (0.1–0.8)	0.5 (0.1–0.8)	0.5 (0.1–0.8)
Mean time in PD3 in years (95% CI)	0 (0–0.1)	0 (0–0.1)	0 (0–0.1)	0 (0–0.1)	0 (0–0.1)	0 (0–0.1)	0 (0–0.1)	0 (0–0.1)	0 (0–0.1)	0 (0–0.1)	0 (0–0.1)
Mean time in PF4 in years (95% CI)	0.4 (0–0.8)	0.4 (0–0.8)	0.4 (0–0.8)	0.4 (0–0.8)	0.4 (0–0.8)	0.4 (0–0.8)	0.4 (0–0.8)	0.4 (0–0.8)	0.4 (0–0.8)	0.4 (0–0.8)	0.4 (0–0.8)
2-y survival	37%	46%	46%	46%	35%	44%	44%	44%	52%	52%	52%
5-y survival	21%	26%	26%	26%	20%	25%	24%	25%	31%	31%	31%

CI, confidence interval; PF1, progression free during or after treatment line 1; PD1, progressive disease after treatment line 1; PF2, progression free during or after treatment line 2; PD2, progressive disease after treatment line 2; PF3, progression free during or after treatment line 3; PD3, progressive disease after treatment line 3; PF4, progression free during and progressive disease after subsequent therapies.

The life expectancy and mean time across health states are derived from the probabilistic sensitivity analyses.

a Outcomes of current treatment (mix [observed in clinical practice]) did not require an adjustment of the hazard ratios with equation 1.

b Real-world treatment mix refers to the total mix of third-line therapies observed in clinical practice (regardless of the type of first- and second-line treatment).

Treatment sequences with nivolumab plus ipilimumab in the first line followed by a BRAF plus MEK inhibitor in the second line yielded the highest life expectancy: 11.9 to 12.0 years in patients with favorable prognostic factors and 7.6 to 7.7 years in patients with intermediate prognostic factors, respectively. Differences are relatively small, with sequences consisting of a BRAF plus MEK inhibitor in the first line followed by nivolumab plus ipilimumab in the second line; the mean life-years are estimated to be 11.4 to 11.5 years in patients with favorable prognostic factors and 7.3 to 7.4 years in patients with intermediate prognostic factors, respectively. Incremental life-years are 0.5 year in patients with favorable prognostic factors and 0.3 year in patients with intermediate prognostic factors, respectively.

Table 3 shows the life expectancy of patients without a BRAF mutation and favorable or intermediate prognostic factors and the impact of different treatment sequences. Life expectancy ranged from 7.8 to 10.1 years in patients with favorable prognostic factors and from 6.1 to 8.7 years in patients with intermediate prognostic factors when they are treated with treatment sequences consisting of immunotherapies, which was substantially higher than the life-years observed for chemotherapy only (3.0 and 1.6 life-years in patients with favorable and intermediate prognostic factors, respectively). Starting treatment with nivolumab plus ipilimumab yielded the highest life expectancy. Differences in life-years compared with a sequence consisting of nivolumab in the first line followed by ipilimumab in the second line amount to 1.9 and 2.1 years in patients with favorable and intermediate prognostic factors, respectively.

**Table 3:** Life Expectancy in Years and Mean Time Spent in Different Health States of Patients without a BRAF Mutation Stratified by Prognostic Factors: Results of the Base Case

First-Line Treatment	Mix (Observed in Clinical Practice)^a^	Chemotherapy	Nivolumab			Pembrolizumab			Nivolumab plus Ipilimumab
Second-Line Treatment	Mix (Observed in Clinical Practice)^a^	Chemotherapy	Real-World Treatment Mix^b^	Ipilimumab	Nivolumab plus Ipilimumab	Real-World Treatment Mix^b^	Ipilimumab	Nivolumab plus Ipilimumab	Real-World Treatment Mix^b^
Patients with favourable prognostic factors									
Life expectancy in years (95% CI)	7.1 (5.1–9.1)	3 (2.2–3.7)	8.6 (5.7–11.5)	8.2 (5.2–11.3)	9.9 (6.7–13.1)	8.2 (5.1–11.3)	7.8 (4.5–11.1)	9.5 (6.1–12.9)	10.1 (6.4–13.9)
Mean time in PF1 in years (95% CI)	4.1 (2.2–6)	1.5 (0.9–2.1)	5.6 (2.7–8.5)	5.6 (2.7–8.5)	5.6 (2.7–8.5)	5.2 (2.2–8.2)	5.2 (2.2–8.2)	5.2 (2.2–8.2)	7 (3.3–10.8)
Mean time in PD1 in years (95% CI)	1.1 (0.7–1.6)	0.5 (0.3–0.8)	1.3 (0.7–1.9)	1.3 (0.7–1.9)	1.3 (0.7–1.9)	1.2 (0.5–2)	1.2 (0.5–2)	1.2 (0.5–2)	1.4 (0.7–2.1)
Mean time in PF2 in years (95% CI)	0.8 (0.4–1.2)	0.3 (0.2–0.4)	0.8 (0.4–1.2)	0.5 (0.2–0.8)	1.7 (0.7–2.7)	0.8 (0.4–1.2)	0.5 (0.2–0.8)	1.7 (0.7–2.8)	0.7 (0.4–1.1)
Mean time in PD2 in years (95% CI)	0.6 (0.3–0.9)	0.3 (0.1–0.4)	0.6 (0.2–0.9)	0.5 (0.1–0.9)	0.8 (0.3–1.4)	0.6 (0.2–0.9)	0.5 (0–0.9)	0.9 (0.2–1.5)	0.5 (0.2–0.9)
Mean time in PF3 in years (95% CI)	0.4 (0–0.9)	0.3 (0–0.7)	0.4 (0–0.8)	0.4 (0–0.8)	0.5 (0–0.9)	0.4 (0–0.8)	0.4 (0–0.8)	0.5 (0–0.9)	0.4 (0–0.8)
2-y survival	0.59	0.29	0.66	0.63	0.72	0.64	0.61	0.69	0.72
5-y survival	0.35	0.13	0.43	0.4	0.49	0.4	0.37	0.47	0.49
Patients with intermediate prognostic factors									
Life expectancy in years (95% CI)	5.6 (4.6–6.7)	1.6 (1.3–2)	6.9 (5.1–8.8)	6.6 (4.7–8.6)	7.8 (5.7–10)	6.4 (4–8.8)	6.1 (3.6–8.6)	7.3 (4.6–10.1)	8.7 (6.2–11.2)
Mean time in PF1 in years (95% CI)	4 (3.1–5)	1.1 (0.8–1.4)	5.2 (3.4–7)	5.2 (3.4–7)	5.2 (3.4–7)	4.7 (2.5–6.9)	4.7 (2.5–6.9)	4.7 (2.5–6.9)	6.8 (4.4–9.2)
Mean time in PD1 in years (95% CI)	0.7 (0.3 – 1.1)	0.2 (0.1–0.4)	0.8 (0.2–1.5)	0.8 (0.2–1.5)	0.8 (0.2–1.5)	0.8 (0.1–1.5)	0.8 (0.1–1.5)	0.8 (0.1–1.5)	1 (0.2–1.8)
Mean time in PF2 in years (95% CI)	0.7 (0.2–1.2)	0.2 (0.1–0.3)	0.7 (0.2–1.2)	0.4 (0.1–0.8)	1.6 (0.5–2.6)	0.7 (0.2–1.2)	0.4 (0–0.8)	1.6 (0.5–2.7)	0.7 (0.2–1.2)
Mean time in PD2 in years (95% CI)	0.1 (0–0.2)	0 (0–0.1)	0.1 (0–0.2)	0.1 (0–0.2)	0.2 (0–0.4)	0.1 (0–0.2)	0.1 (0–0.2)	0.2 (0–0.4)	0.1 (0–0.2)
Mean time in PF3 in years (95% CI)	0.1 (0–0.2)	0.1 (0–0.2)	0.1 (0–0.2)	0.1 (0–0.2)	0.1 (0–0.3)	0.1 (0–0.2)	0.1 (0–0.2)	0.1 (0–0.3)	0.1 (0–0.2)
2-years survival	0.41	0.12	0.48	0.45	0.53	0.45	0.42	0.50	0.56
5-years survival	0.25	0.06	0.30	0.29	0.35	0.28	0.26	0.32	0.38

CI, confidence interval; PF1, progression free during or after treatment line 1; PD1, progressive disease after treatment line 1; PF2, progression free during or after treatment line 2; PD2, progressive disease after treatment line 2; PF3, progression free during or after treatment line 3; PD3, progressive disease after treatment line 3; PF4, progression free during and progressive disease after subsequent therapies.

The life expectancy and mean time across health states are derived from the probabilistic sensitivity analyses.

a Outcomes of current treatment (mix [observed in clinical practice]) did not require an adjustment of the hazard ratios with equation 1.

b Real-world treatment mix refers to the total mix of second-line therapies observed in clinical practice (regardless of the type of first-line treatment).

### Impact of Alternative Assumptions in Scenario Analyses

Table 4 illustrates the impact of different assumptions on life expectancy of patients with a BRAF mutation, stratified by prognostic factors, for all treatment sequences. Here, we describe the results for patients with intermediate prognostic factors.

**Table 4:** Life Expectancy (in Years) in the Base Case and Different Scenarios of Patients with a BRAF Mutation Stratified by Prognostic Factors

First-Line Treatment	Mix (Observed in Clinical Practice)^a^	Chemotherapy	Dabrafenib plus Trametinib			Vemurafenib plus Cobimetinib			Encorafenib plus Binimetinib		
Second-Line Treatment	Mix (Observed in Clinical Practice)^a^	Chemotherapy	Nivolumab	Pembrolizumab	Ipilimumab plus Nivolumab	Nivolumab	Pembrolizumab	Ipilimumab plus Nivolumab	Nivolumab	Pembrolizumab	Ipilimumab plus Nivolumab
Third-Line Treatment	Mix (Observed in Clinical Practice)^a^	Chemotherapy	Real-World Treatment Mix^b^	Real-World Treatment Mix^b^	Real-World Treatment Mix^b^	Real-World Treatment Mix^b^	Real-World Treatment Mix^b^	Real-World Treatment Mix^b^	Real-World Treatment Mix^b^	Real-World Treatment Mix^b^	Real-World Treatment Mix^b^
Patients with favorable prognostic factors											
Base case	9.4 (8.2–10.6)	3.6 (2.9–4.3)	10.7 (9–12.4)	10.4 (8.6–12.2)	11.5 (9.6–13.4)	10.6 (8.9–12.3)	10.3 (8.5–12.2)	11.4 (9.5–13.3)	10.7 (9–12.4)	10.4 (8.6–12.2)	11.5 (9.6–13.4)
Scenario 1	9.4 (8.2–10.6)	3.6 (2.9–4.3)	10.4 (8.7–12.1)	10.1 (8.3–12)	11.2 (9.4–13.1)	10.3 (8.7– 11.9)	10.1 (8.3–11.9)	11.1 (9.3–13)	10.4 (8.7–12.1)	10.1 (8.3–12)	11.2 (9.4–13.1)
Scenario 2	9.4 (8.2–10.6)	3.6 (2.9–4.3)	11.1 (9.3–12.9)	10.9 (8.9–12.9)	11.9 (9.9–13.8)	11 (9.1–12.8)	10.8 (8.7–12.8)	11.7 (9.7–13.8)	11.1 (9.3–12.9)	10.9 (8.9–12.9)	11.9 (9.9–13.8)
Scenario 3	9.4 (8.2–10.6)	3.6 (2.9–4.3)	11.5 (9.4–13.7)	11.2 (8.5–13.9)	13.2 (10.5–15.8)	11.4 (9.2–13.6)	11.1 (8.3–13.8)	13 (10.3–15.8)	11.5 (9.4–13.7)	11.2 (8.5–13.9)	13.2 (10.5–15.8)
Scenario 4	4.5 (4.2 – 4.9)	2.1 (1.9 – 2.3)	5 (4.5–5.4)	4.9 (4.4–5.4)	5.2 (4.8–5.7)	5 (4.5–5.4)	4.9 (4.4–5.4)	5.2 (4.7–5.7)	5 (4.5–5.4)	4.9 (4.4–5.4)	5.2 (4.8–5.7)
Patients with intermediate prognostic factors											
Base case	6.1 (4.8–7.3)	1.7 (1.2–2.3)	6.8 (5.1–8.5)	6.7 (5–8.4)	7.4 (5.6–9.2)	6.7 (5.1–8.3)	6.6 (4.9–8.2)	7.3 (5.5–9.1)	6.8 (5.1–8.5)	6.7 (5–8.4)	7.4 (5.6–9.2)
Scenario 1	6.1 (4.8–7.3)	1.7 (1.2–2.3)	6.6 (5–8.1)	6.4 (4.8–8)	7.2 (5.4–8.9)	6.5 (4.9–8.1)	6.4 (4.7–8)	7.1 (5.3–8.9)	6.6 (5–8.1)	6.4 (4.8–8)	7.2 (5.4–8.9)
Scenario 2	6.1 (4.8–7.3)	1.7 (1.2–2.3)	7.3 (5.4–9.1)	7.1 (5.3–9)	7.8 (5.9–9.8)	7.1 (5.3–8.9)	7 (5.2–8.9)	7.7 (5.8–9.7)	7.3 (5.4–9.1)	7.1 (5.3–9)	7.8 (5.9–9.8)
Scenario 3	6.1 (4.8–7.3)	1.7 (1.2–2.3)	7 (5.2–8.8)	6.9 (4.9–8.8)	7.9 (5.8–10.1)	6.9 (5.1–8.7)	6.7 (4.8–8.7)	7.8 (5.7–10)	7 (5.2–8.8)	6.9 (4.9–8.8)	7.9 (5.8–10.1)
Scenario 4	3.3 (2.9–3.7)	1.2 (0.9–1.5)	3.6 (3–4.2)	3.5 (3–4.1)	3.8 (3.2–4.4)	3.5 (3–4.1)	3.5 (2.9–4.1)	3.8 (3.2–4.4)	3.6 (3–4.2)	3.5 (3–4.1)	3.8 (3.2–4.4)
First-Line Treatment	Nivolumab				Pembrolizumab				Nivolumab plus Ipilimumab		
Second-Line Treatment	Ipilimumab	Dabrafenib plus Trametinib	Vemurafenib plus Cobimetinib	Encorafenib plus Binimetinib	Ipilimumab	Dabrafenib plus Trametinib	Vemurafenib plus Cobimetinib	Encorafenib plus Binimetinib	Dabrafenib plus Trametinib	Vemurafenib plus Cobimetinib	Encorafenib plus Binimetinib
Third-Line Treatment	Real-World Treatment Mix^b^	Real-World Treatment Mix^b^	Real-World Treatment Mix^b^	Real-World Treatment Mix^b^	Real-World Treatment Mix^b^	Real-World Treatment Mix^b^	Real-World Treatment Mix^b^	Real-World Treatment Mix^b^	Real-World Treatment Mix^b^	Real-World Treatment Mix^b^	Real-World Treatment Mix^b^
Patients with favorable prognostic factors											
Base case	9 (7.2–10.8)	10.8 (9.2–12.5)	10.7 (9.1–12.4)	10.8 (9.2–12.5)	8.6 (6.4–10.8)	10.4 (8.5–12.4)	10.3 (8.3–12.4)	10.4 (8.5–12.4)	12 (10–14)	11.9 (9.9–13.9)	12 (10–14)
Scenario 1	9 (7.2–10.8)	10.6 (8.9–12.2)	10.5 (8.9–12.2)	10.6 (8.9–12.2)	8.6 (6.5–10.8)	10.2 (8.2–12.2)	10.2 (8.2–12.1)	10.2 (8.2–12.2)	11.7 (9.7–13.7)	11.7 (9.7–13.6)	11.7 (9.7–13.7)
Scenario 2	8.9 (7.1–10.8)	11.1 (9.4–12.9)	11 (9.2–12.8)	11.1 (9.4–12.9)	8.6 (6.3–10.9)	10.8 (8.7–12.9)	10.7 (8.6–12.8)	10.8 (8.7–12.9)	12.2 (10.2–14.3)	12.1 (10–14.3)	12.2 (10.2–14.3)
Scenario 3	10.8 (8.2–13.4)	12.2 (9.7–14.8)	12.2 (9.7–14.7)	12.2 (9.7–14.8)	10.2 (6.6–13.9)	11.6 (8–15.3)	11.6 (8–15.2)	11.6 (8–15.3)	14.5 (11.2–17.7)	14.4 (11.2–17.7)	14.5 (11.2–17.7)
Scenario 4	4.3 (3.7–4.9)	5 (4.5–5.5)	5 (4.5–5.5)	5 (4.5–5.5)	4.1 (3.4–4.9)	4.9 (4.3–5.5)	4.9 (4.3–5.4)	4.9 (4.3–5.5)	5.4 (4.8–5.9)	5.3 (4.8–5.9)	5.4 (4.8–5.9)
Patients with intermediate prognostic factors											
Base case	5.4 (3.8–6.9)	6.5 (4.9–8.1)	6.5 (4.9–8)	6.5 (4.9–8.1)	5.1 (3.3–6.9)	6.2 (4.3–8)	6.1 (4.3–8)	6.2 (4.3–8)	7.7 (5.7–9.6)	7.6 (5.7–9.6)	7.7 (5.7–9.6)
Scenario 1	5.4 (3.9–6.8)	6.4 (4.9–7.9)	6.4 (4.8–7.9)	6.4 (4.9–7.9)	5.1 (3.3–6.9)	6.1 (4.3–7.9)	6 (4.2–7.8)	6.1 (4.3–7.9)	7.5 (5.7–9.4)	7.5 (5.6–9.4)	7.5 (5.7–9.4)
Scenario 2	5.4 (3.9–6.9)	6.8 (5.1–8.4)	6.7 (5.1–8.3)	6.8 (5.1–8.4)	5.1 (3.3–6.9)	6.5 (4.5–8.4)	6.4 (4.5–8.3)	6.5 (4.5–8.4)	8 (5.9–10)	7.9 (5.9–9.9)	8 (5.9–10)
Scenario 3	6.2 (4.3–8.1)	7.1 (5.1–9.2)	7.1 (5.1–9.2)	7.1 (5.1–9.2)	5.8 (3.3–8.3)	6.7 (4–9.4)	6.7 (4–9.3)	6.7 (4–9.4)	9 (6.2–11.8)	9 (6.2–11.8)	9 (6.2–11.8)
Scenario 4	3 (2.4–3.5)	3.5 (2.9–4)	3.5 (2.9–4)	3.5 (2.9–4)	2.8 (2.1–3.6)	3.3 (2.6–4)	3.3 (2.6–4)	3.3 (2.6–4)	3.9 (3.3–4.6)	3.9 (3.3–4.5)	3.9 (3.3–4.6)

All estimates in this table are derived from probabilistic sensitivity analyses.

a Outcomes of current treatment (mix [observed in clinical practice]) did not require an adjustment of the hazard ratios with equation 1.

b Real-world treatment mix refers to the total mix of third-line therapies observed in clinical practice (regardless of the type of first- and second-line treatment).

Alternative durations of the relative treatment effect of targeted therapies regarding PFS have a relatively small impact on estimates of life expectancy. For example, assuming a duration of relative treatment effect of targeted therapies regarding PFS of 0.25 years instead of 0.5 years (scenario 1) decreases the mean life expectancy from 7.4 to 7.2 years in patients receiving dabrafenib plus trametinib or encorafenib plus binimetinib in the first line followed by nivolumab plus ipilimumab in the second line. This alternative assumption also influences the outcomes of treatment sequences with targeted therapy as second-line treatment. For example, in patients receiving nivolumab plus ipilimumab in the first line followed by dabrafenib plus trametinib or encorafenib plus binimetinib in the second line, life expectancy decreases from 7.7 to 7.5 years. Incremental life-years hardly change.

Assuming a relative treatment effect duration of 1 year instead of 0.5 years (scenario 2) increases life expectancy from 7.4 to 7.8 years in patients receiving dabrafenib plus trametinib or encorafenib plus binimetinib in the first line followed by nivolumab plus ipilimumab in the second line. In patients receiving nivolumab plus ipilimumab in the first line followed by dabrafenib plus trametinib or encorafenib plus binimetinib in in the second line, life expectancy increases from 7.7 to 8.0 years. Incremental life-years decrease from 0.3 year to 0.2 year.

In the base case, we assumed that treatments have an impact on OS only during the line in which the therapy is provided to patients. An alternative assumption could be that the duration of the relative treatment effect of immunotherapies regarding OS holds beyond the line of therapy and hence that immunotherapies in a previous line positively affect the time to death in later lines (scenario 3). Changing this assumption had a larger impact; life expectancy increases from 7.4 to 7.9 years in patients receiving dabrafenib plus trametinib or encorafenib plus binimetinib in the first line followed by nivolumab plus ipilimumab in the second line. Life expectancy increases from 7.7 to 9.0 years in patients receiving nivolumab plus ipilimumab in the first line followed by dabrafenib plus trametinib or encorafenib plus binimetinib in the second line. Incremental life-years increase from 0.3 year to 1.1 years.

Reducing the time horizon of the model from lifetime to 10 years had a relatively large impact on life expectancy since a shorter time horizon restricts the life expectancy for all patients to a maximum of 10 years, but it has a very small impact on incremental life-years (scenario 4).

The impact of different assumptions on life expectancy of patients without a BRAF mutation, stratified by prognostic factors, for all treatment sequences is presented in Supplementary Table 6.1.

## Discussion

This study showed the development of a model spanning multiple lines of treatment to estimate the long-term health benefits of treatments and treatment sequences for patients with advanced melanoma with and without a BRAF mutation. It showed that the effectiveness of treatment sequences depends on the place of different therapies within a sequence and that the absolute outcomes of treatment sequences differ for patients with favorable and intermediate prognostic factors. Furthermore, it showed the outcomes of treatment sequences under alternative assumptions regarding the duration of relative treatment effects. With the model, we estimated outcomes of treatment sequences up to 3 lines of systemic treatment, which contributes in filling the current research gap in outcomes of treatment sequences^15,28^ and the need for the development of decision models that enable such comparisons.^3^ By incorporating flexibility regarding assumptions about the impact of subsequent treatment on life expectancy, we aim to overcome some of the previously identified shortcomings.^22^

A key feature of our model is that transitions describing disease progression, time to next treatment, and mortality were estimated from Dutch real-world data while the relative effectiveness of treatments was derived from an NMA based on RCTs. An NMA provides a simultaneous comparison of multiple treatments by combining direct and indirect relative efficacy evidence from RCTs and has been used in previous treatment sequence models^33,34^ in the absence of head-to-head comparisons. Using the NMA instead of real-world data to inform the relative effectiveness of treatments ensured that estimates of treatment effects are based on a high level of evidence. The systematic literature review and NMA did not use any exclusion criteria with respect to region. The RCTs included are therefore international and involve participants from various countries across multiple regions. By using real-world data alongside trial data, we aimed to ensure the generalizability of the outcomes of the model to patients treated in clinical practice. Since we used real-world data from the Netherlands, long-term health benefits of treatment sequences in patients in other countries might be different. Nevertheless, we believe this study offers valuable insights to clinicians and policy makers in other countries, even if only regarding the ranking of treatment sequences providing most health benefits.

Our estimates of the life expectancy of patients treated as observed in clinical practice aligned with the outcomes from the French MelBase cohort.^25^ Using a 10-years time horizon, Kandel et al.^25^ estimated an average life expectancy of 3.6 years. Although the patient populations were defined slightly differently, these results align with our predictions that ranged from 3.3 to 4.5 years using a 10-years time horizon. Furthermore, we found relatively large differences between the median and mean OS based on lifetime horizon, but this aligns with clinical experience and existing research.^35^

A comparison of our long-term estimates with trial data is hindered by insufficient follow-up of patients participating in a trial, different subsequent treatments that were available at the time of the studies, and differences in patient characteristics. Nonetheless, long-term estimates of OS are presented for treatment with a combination of ipilimumab and nivolumab. We estimated a 5-years OS of 45% and 31% for patients with a BRAF mutation and 49% and 38% for patients without a BRAF mutation with favorable and intermediate prognostic factors, respectively. These estimates are all below the outcomes of the phase III trial of 60% and 38% for patients with normal LDH and for patients with elevated LDH, regardless of BRAF mutation.^36^ Estimates of long-term OS from the phase III trial were confirmed in recent publications.^37^ Lower survival compared with the outcomes of the phase III trial was expected, because we adjusted the outcomes for the patient and disease characteristics that we observed in clinical practice.^38^

Although many treatment sequences were included in the analysis, the treatment landscape is subject to constant change, which should be considered when interpreting the outcomes of the model in terms of life expectancy. Some of the treatment sequences that were evaluated in the current study are rarely used within current Dutch clinical practice these days, whereas (neo)adjuvant treatments have been introduced recently that were not considered in the current study.

### Limitations

This study was subject to a number of limitations. First, the scientific evidence on many parameters in the model is uncertain (e.g., long-term effectiveness of treatments) or lacking altogether (e.g., a lack of trials with a sole focus on previously treated patients). For example, due to the lack of data to quantify the impact of the effect of prior treatments, we assumed that the relative effect of treatment found in the NMA was representative for the effect of a treatment in a sequence, regardless of treatment line. Hence, the relative treatment effect is assumed to be independent of the type and number of previous therapies. The results from the NMA that we used to inform our model were based on RCTs regarding patients who were not previously treated with novel treatments.^11^ The lack of (randomized) evidence to support the relative effectiveness of treatments in a particular sequence has been identified before.^33,34^ Until now, evidence has been available about the efficacy of only some treatment sequences.^32,39^ This evidence is, however, insufficient to inform our model about the relative effectiveness of treatments in a sequence. For example, the DREAMSeq study showed the outcomes of nivolumab plus ipilimumab in the first line followed by dabrafenib plus trametinib in the second line but did not provide evidence of the effectiveness of dabrafenib plus trametinib in the second line relative to other treatments let alone a quantification of the relative effectiveness accounting for prior treatment. Although we did not adjust the relative effectiveness of treatments with respect to their place in a sequence, we did adjust the initial hazard functions for patient characteristics and prior treatment by estimating these from real-world data on a cohort of patients transitioning from first-line treatment to subsequent treatments, following recommendations for modeling effectiveness in oncology treatment sequence models.^34^

Second, for some subgroups, long-term survival might have been underestimated, especially for patients with a BRAF mutation with intermediate prognostic factors. Real-world data showed a plateau in the Kaplan-Meier survival curve from month 39, while this plateau starts later and at a lower level in the modeled outcomes. However, it should be noted that there was censoring during the final follow-up period. Alternative methods for survival analyses, such as flexible parametric survival methods incorporating splines or models that enforce cure proportions, might be a solution to this limitation. Because we believe that for the other subgroups, the model fits the data relatively well, we did not explore those solutions in this study.

Third, the cohort modeling approach we have used poses some limitations to the flexibility of our model. We defined subgroups of patients based on their prognostic factors at the start of a first-line therapy, and we assumed that those patients who survive and start a new treatment line (i.e., enter PF2 or PF3) are eligible to receive a second- or third-line treatment. Furthermore, baseline characteristics were taken into account as covariates in the hazard functions for all transitions between health states, while patient and disease characteristics at the start of a second- or third-line treatment might be better predictors for the hazard rates over time from subsequent health states (i.e., PF2 or PF3). A patient-level simulation model would have been able to capture changes in patient and disease characteristics but requires data to inform changes of these characteristics as a function of time and treatment and in relation to the outcomes of our model (i.e., life-years). Therefore, we believe that the advantages of a cohort model, such as ease of model development, limited running time, and the agreement with commonly used structures of decision models in cancer, outweigh the disadvantages in our case. Although a patient-level simulation model would also provide a convenient structure to take into account duration dependency (i.e., changing risks of events over time, specifically after an event), we took into account duration dependency by incorporating tunnel states within our model.

Finally, although the most extensive Dutch database for melanoma patients was used which allowed us to overcome some of the challenges identified previously,^34^ we encountered missing data in baseline characteristics. As a consequence, not all patients could be included in the analyses. Patient numbers in especially the later transitions after two lines of therapy were small. Estimates of these hazard functions are therefore more uncertain. However, since treatments in these lines are the same across the sequences we evaluated, this did not have an impact on differences in outcomes between treatment sequences. Additionally, the number of patients that switch to a next treatment without progression of disease might have been overestimated due to missing data about progression of disease. Since these patients, in the model, move directly from progression-free to next treatment, they do not benefit from any treatment effect, and therefore benefits of treatments that are more effective than the treatment of the population from which the transitions are estimated might have been somewhat underestimated.

## Conclusions

This study provides valuable insights into how to develop a decision model to estimate the long-term health benefits of treatment sequences. We show how to use real-world data and data from clinical trials to obtain the most benefit from the advantages of both data sources. The model is flexible because it can accommodate different treatments and treatment sequences. Also, the duration of treatment effects can be adjusted as well as the transitions that are influenced by treatment. Furthermore, model inputs can easily be adapted when new data become available. Research regarding the duration of treatment effects as well as the effectiveness of treatments within a sequence should be a key priority. To support decision making, the model should be expanded to capture quality-adjusted life-years and costs to estimate the cost-effectiveness of treatment sequences.

## Supplemental Material

sj-docx-1-mdm-10.1177_0272989X251319338 – Supplemental material for Development of a Decision Model to Estimate the Outcomes of Treatment Sequences in Advanced Melanoma Supplemental material, sj-docx-1-mdm-10.1177_0272989X251319338 for Development of a Decision Model to Estimate the Outcomes of Treatment Sequences in Advanced Melanoma by Saskia de Groot, Hedwig M. Blommestein, Brenda Leeneman, Carin A. Uyl-de Groot, John B. A. G. Haanen, Michel W. J. M. Wouters, Maureen J. B. Aarts, Franchette W. P. J. van den Berkmortel, Willeke A. M. Blokx, Marye J. Boers-Sonderen, Alfons J. M. van den Eertwegh, Jan Willem B. de Groot, Geke A. P. Hospers, Ellen Kapiteijn, Olivier J. van Not, Astrid A. M. van der Veldt, Karijn P. M. Suijkerbuijk and Pieter H. M. van Baal in Medical Decision Making
