# Supplementary material for: Development of a Decision Model to Estimate the Outcomes of Treatment Sequences in Advanced Melanoma
Source: Med Decis Making. 2025 Feb 22;45(3):302–17. doi: 10.1177/0272989X251319338 (PMC11894896; doi:10.1177/0272989X251319338)
Supplement: sj-docx-1-mdm-10.1177_0272989X251319338 – Supplemental material for Development of a Decision Model to Estimate the Outcomes of Treatment Sequences in Advanced Melanoma [file sj-docx-1-mdm-10.1177_0272989X251319338.docx]

# Online supplement

# Descriptives of patient population

Figure 1.1 Patients included in the analysis to estimate parameters of the model


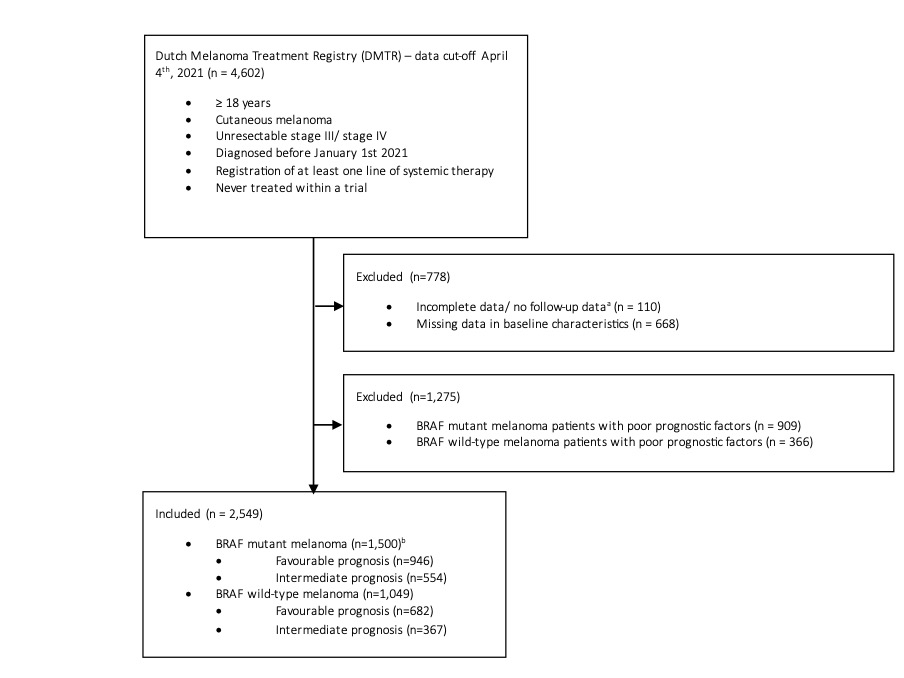


^a^ Patients with incomplete data regarding key variables were excluded (i.e., the start date of first-line therapy, the stop date, the date a patient died and the date of the last visit to the hospital [which was also used to create the date of progression of disease]).

^b^ The number of patients with a BRAF mutation might seem relatively high compared to the number of patients without a BRAF mutation. A possible explanation is that we did not include patients without the registration of at least one line of systemic therapy. Among these, there are more patients without a BRAF mutation.

Table 1.1 Baseline patient and disease characteristics

|  | Advanced melanoma with a BRAF mutation | | Advanced melanoma without a BRAF mutation | |
| --- | --- | --- | --- | --- |
|  | Favourable  prognostic factors  (n=946) | Intermediate prognostic factors  (n=554) | Favourable  prognostic factors  (n=682) | Intermediate prognostic factors  (n=367) |
| **Gender**, *male (%)* | 56% | 57% | 66% | 62% |
| **Age**, *mean*  *median (IQR)* | 60  61 (51-70) | 61  62 (52-71) | 66  69 (58-75) | 67  69 (59-76) |
| **LDH**, *n (%)*      ≤1 ULN       >1 ULN - ≤2 ULN       >2 ULN | 946 (100%)  -  - | 181 (33%)  373 (67%)  - | 682 (100%)  -  - | 100 (27%)  267 (73%)  - |
| **ECOG**, *n (%)*      0      1      ≥2 | 648 (69%)  298 (32%)  - | 321 (58%)  233 (42%)  - | 466 (68%)  216 (32%)  - | 187 (51%)  180 (49%)  - |
| **M-stage (AJCC7)**, *n (%)*       M0-M1b       M1c  Unknown | 430 (45%)  513 (54%)  3 (0.3%) | 18 (3%)  536 (97%)  - | 320 (47%)  356 (52%)  - | 14 (4%)  353 (96%)  - |
| **Brain metastases**, *n (%)*       No       Yes, asymptomatic       Yes, symptomatic | 946 (100%)  -  - | 284 (51%)  270 (49%)  - | 682 (100%)  -  - | 217 (59%)  150 (41%)  - |

Abbreviations: AJCC, American Joint Committee on Cancer; ECOG, Eastern Cooperative Oncology Group; IQR, interquartile range; LDH, lactate dehydrogenase; ULN, upper limit of normal.

# Treatment characteristics by line

Table 2.1 Treatment by line

|  | **Advanced melanoma with a BRAF mutation** | | **Advanced melanoma without a BRAF mutation** | |
| --- | --- | --- | --- | --- |
|  | **Favourable** | **Intermediate** | **Favourable** | **Intermediate** |
| **Treatment line 1** | **N=946** | **N=554** | **N=682** | **N=367** |
| Encorafenib plus binimetinib | 9 (1%) | 11 (2%) | 0 (0%) | 0 (0%) |
| Chemotherapy | 21 (2%) | 3 (1%) | 38 (6%) | 20 (5%) |
| Dabrafenib | 34 (4%) | 28 (5%) | 0 (0%) | 0 (0%) |
| Dabrafenib plus trametinib | 140 (15%) | 143 (26%) | 0 (0%) | 0 (0%) |
| Ipilimumab | 117 (12%) | 27 (5%) | 147 (22%) | 66 (18%) |
| Ipilimumab plus nivolumab | 69 (7%) | 90 (16%) | 49 (7%) | 115 (31%) |
| Nivolumab | 164 (17%) | 60 (11%) | 201 (29%) | 65 (18%) |
| Other | 19 (2%) | 11 (2%) | 13 (2%) | 4 (1%) |
| Pembrolizumab | 195 (21%) | 74 (13%) | 234 (34%) | 96 (26%) |
| Vemurafenib | 165 (17%) | 82 (15%) | 0 (0%) | 1 (0%) |
| Vemurafenib plus cobimetinib | 13 (1%) | 25 (5%) | 0 (0%) | 0 (0%) |
| **Treatment line 2** | **N=544** | **N=320** | **N=235** | **N=86** |
| Encorafenib plus binimetinib | 18 (3%) | 11 (3%) | 0 (0%) | 0 (0%) |
| Chemotherapy | 4 (1%) | 2 (1%) | 12 (5%) | 4 (5%) |
| Dabrafenib | 30 (6%) | 19 (6%) | 0 (0%) | 0 (0%) |
| Dabrafenib plus trametinib | 139 (26%) | 77 (24%) | 0 (0%) | 0 (0%) |
| Ipilimumab | 104 (19%) | 28 (9%) | 78 (33%) | 26 (30%) |
| Ipilimumab plus nivolumab | 33 (6%) | 69 (22%) | 14 (6%) | 3 (3%) |
| Nivolumab | 51 (9%) | 25 (8%) | 36 (15%) | 12 (14%) |
| Other | 21 (4%) | 9 (3%) | 10 (4%) | 6 (7%) |
| Pembrolizumab | 96 (18%) | 41 (13%) | 85 (36%) | 35 (41%) |
| Vemurafenib | 25 (5%) | 20 (6%) | 0 (0%) | 0 (0%) |
| Vemurafenib plus cobimetinib | 23 (4%) | 19 (6%) | 0 (0%) | 0 (0%) |
| **Treatment line 3** | **N=289** | **N=137** | **N=62** | **N=15** |
| Encorafenib plus binimetinib | 12 (4%) | 6 (4%) | 0 (0%) | 0 (0%) |
| Chemotherapy | 2 (1%) | 1 (1%) | 7 (11%) | 4 (27%) |
| Dabrafenib | 16 (6%) | 10 (7%) | 0 (0%) | 0 (0%) |
| Dabrafenib plus trametinib | 86 (30%) | 54 (39%) | 1 (2%) | 0 (0%) |
| Ipilimumab | 22 (8%) | 15 (11%) | 10 (16%) | 1 (7%) |
| Ipilimumab plus nivolumab | 30 (10%) | 11 (8%) | 0 (0%) | 0 (0%) |
| Nivolumab | 19 (7%) | 12 (9%) | 10 (16%) | 3 (20%) |
| Other | 14 (5%) | 3 (2%) | 9 (15%) | 2 (13%) |
| Pembrolizumab | 51 (18%) | 14 (10%) | 25 (40%) | 5 (33%) |
| Vemurafenib | 20 (7%) | 5 (4%) | 0 (0%) | 0 (0%) |
| Vemurafenib plus cobimetinib | 17 (6%) | 6 (4%) | 0 (0%) | 1. (0%) |

# Survival extrapolation

## BRAF-mutated melanoma favourable prognostic factors

In Table 3.1 below we present the summary statistics of the time to event analysis including number of patients included, number of events and median time to event. Table 3.2 present the AIC and Table 3.3 the BIC statistics. Figure 3.1 provides an overview of the fitted parametric distributions and Figure 3.2 an overview of the selected parametric distributions.

Table 3.1 Summary statistics of the time-to-event analysis BRAF-mutant melanoma patients with favourable prognostic factors


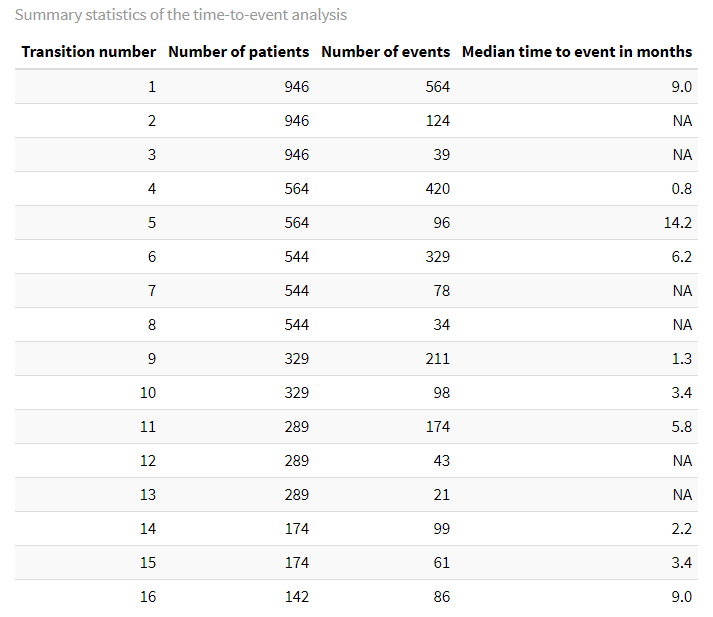


NA: Not available since median time to event was not reached

Note. Transition 1, PF1 -> PD1; Transition 2, PF1 -> PF2; Transition 3, PF1 -> D; Transition 4, PD1 -> PF2; Transition 5, PD1 -> D; Transition 6, PF2 -> PD2; Transition 7, PF2 -> PF3; Transition 8, PF2 -> D; Transition 9, PD2 -> PF3; Transition 10, PD2 -> D; Transition 11, PF3 -> PD3; Transition 12, PF3 -> PF4; Transition 13, PF3 -> D; Transition 14, PD3 -> PF4; Transition 15, PD3 -> D; Transition 16, PF4 -> D.

Table 3.2 AIC values per model per transition for BRAF-mutated melanoma patients with favourable prognostic factors


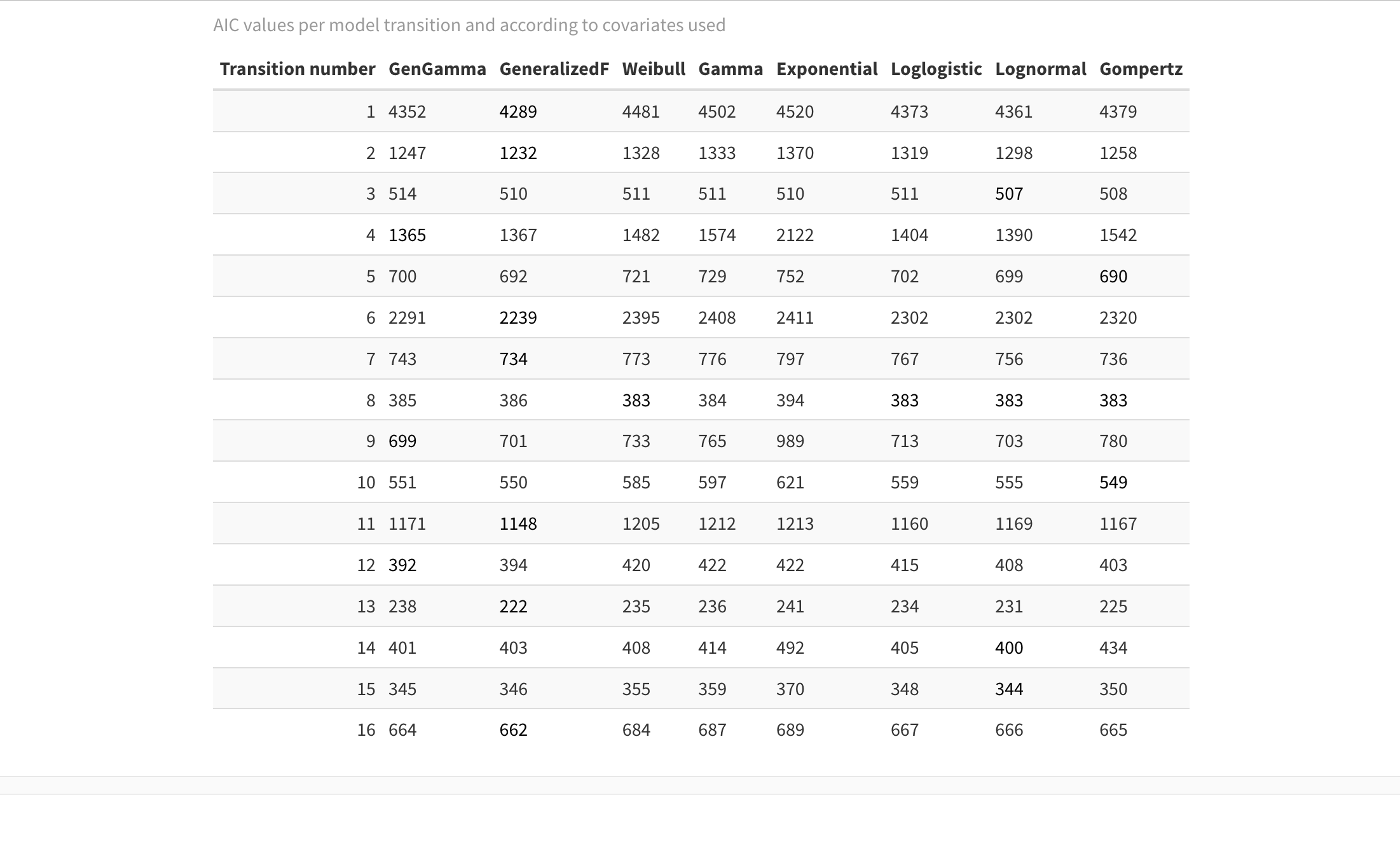


Table 3.3 BIC values per model per transition for BRAF-mutated melanoma patients with favourable prognostic factors


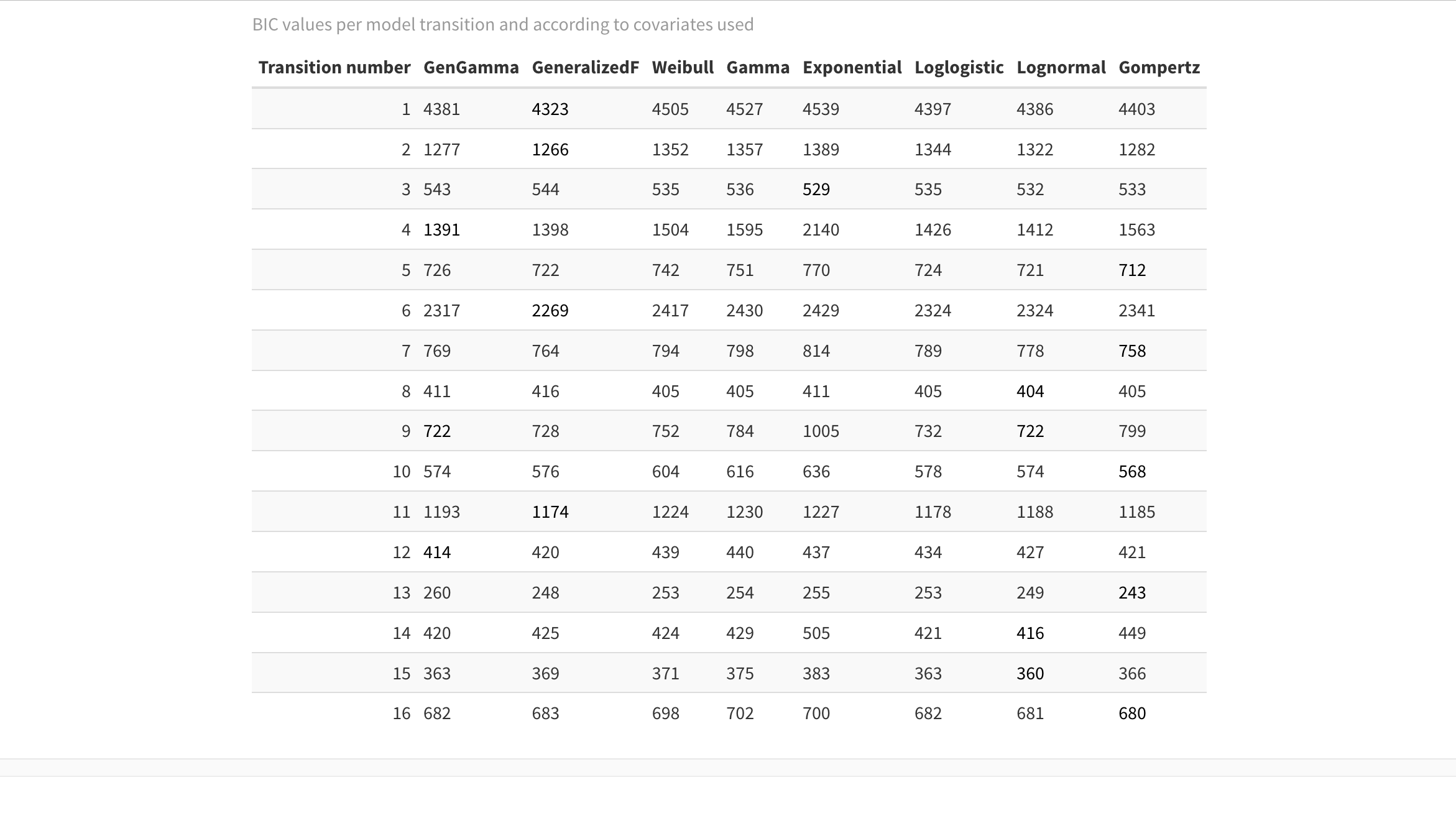


Figure 3.1 Overview of the fitted parametric curves for the transitions, time in months BRAF-mutant melanoma patients with favourable prognostic factors


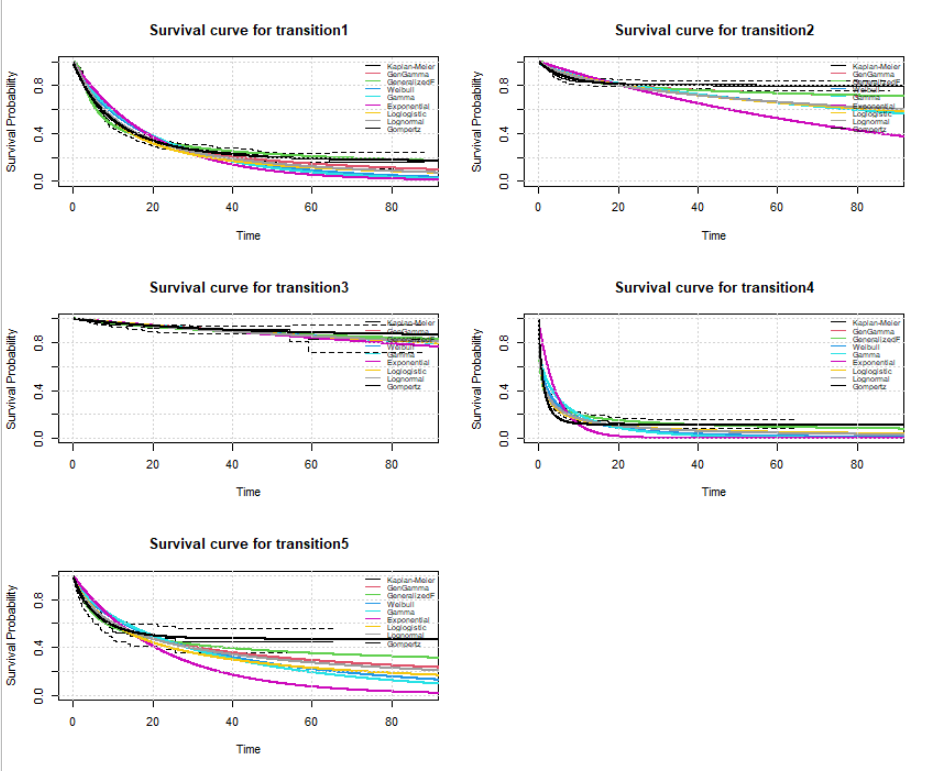


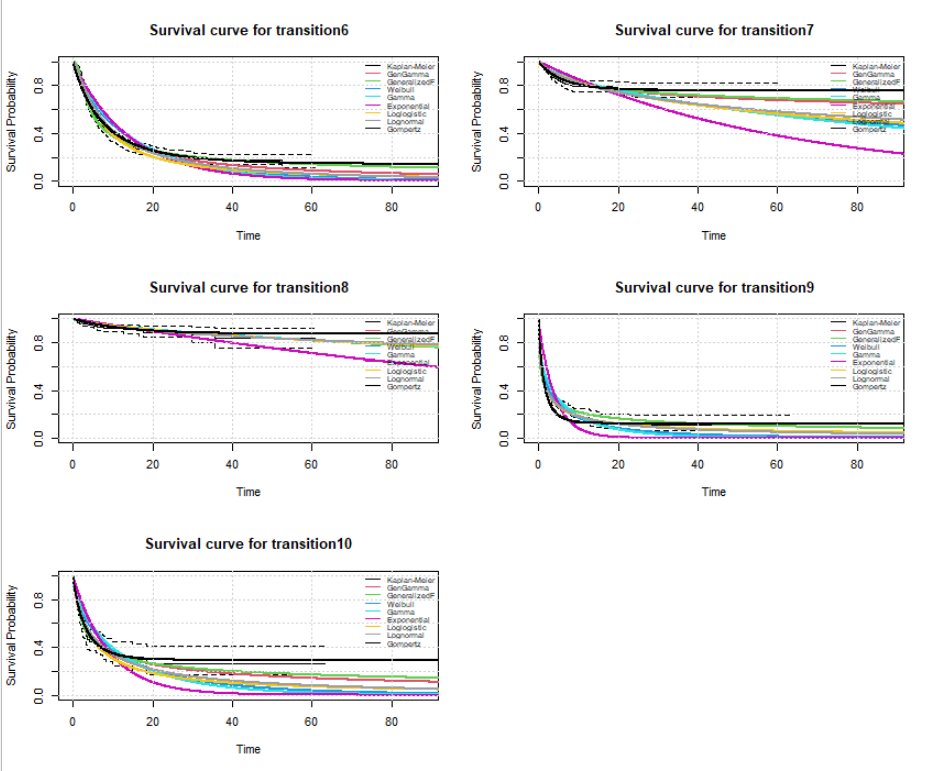


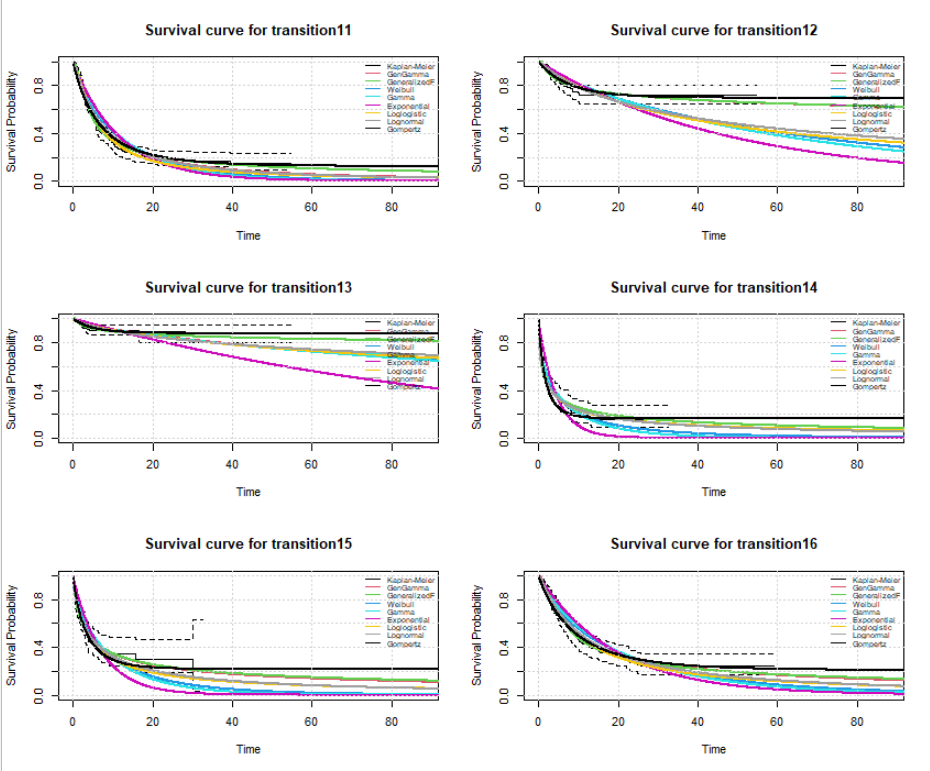


Figure 3.2 Overview of the selected parametric curves for the transitions, time in months, BRAF-mutant melanoma patients with favourable prognostic factors

Transition 1-5 BRAF + Favourable prognostic factors


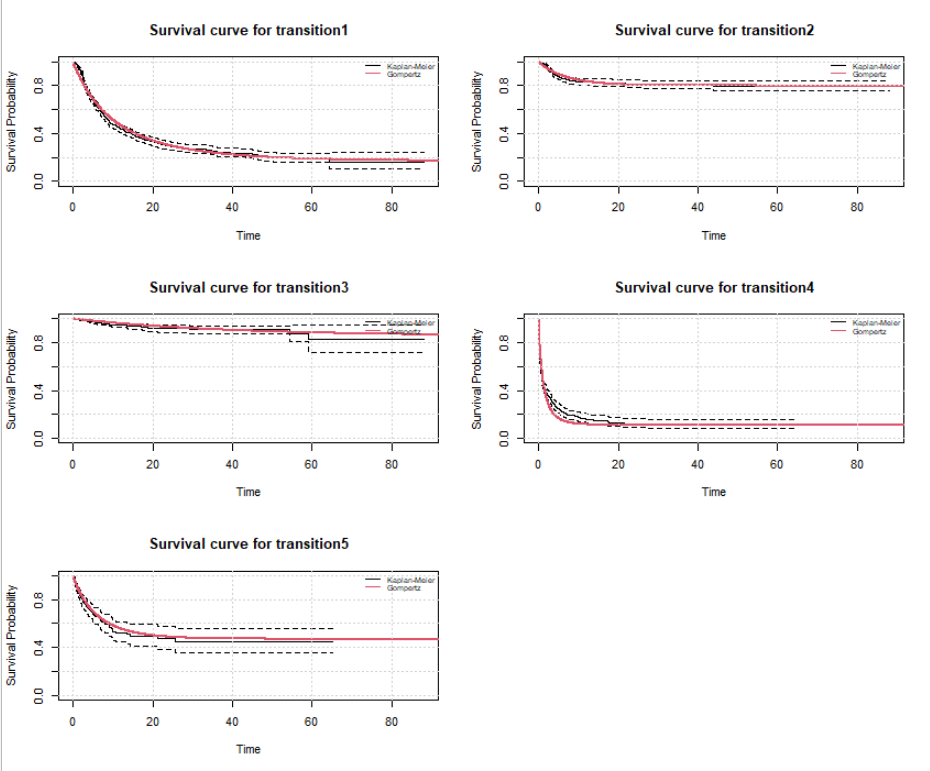


Transition 6-10 BRAF + Favourable prognostic factors


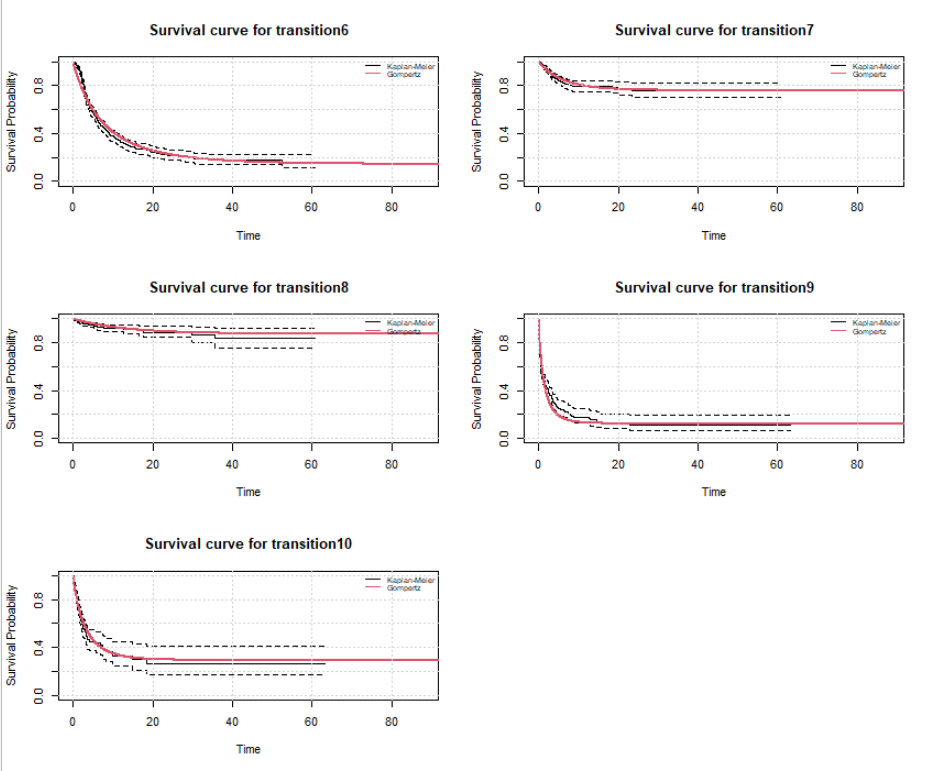


Transition 11-16 BRAF + Favourable prognostic factors


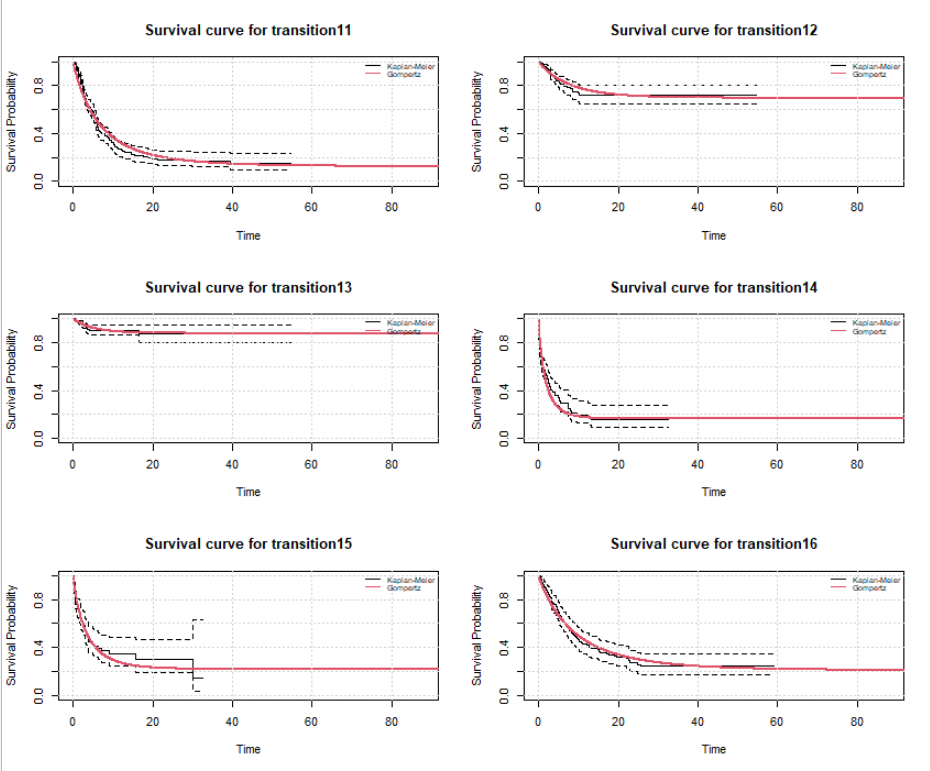


## BRAF-mutated melanoma intermediate prognostic factors

In Table 3.4 below we present the summary statistics of the time to event analysis including number of patients included, number of events and median time to event. Table 3.5 present the AIC and Table 3.6 the BIC statistics. Figure 3.3 provides an overview of the fitted parametric distributions and Figure 3.4 an overview of the selected parametric distributions.

Table 3.4 Summary statistics of the time-to-event analysis BRAF-mutant melanoma patients with intermediate prognostic factors


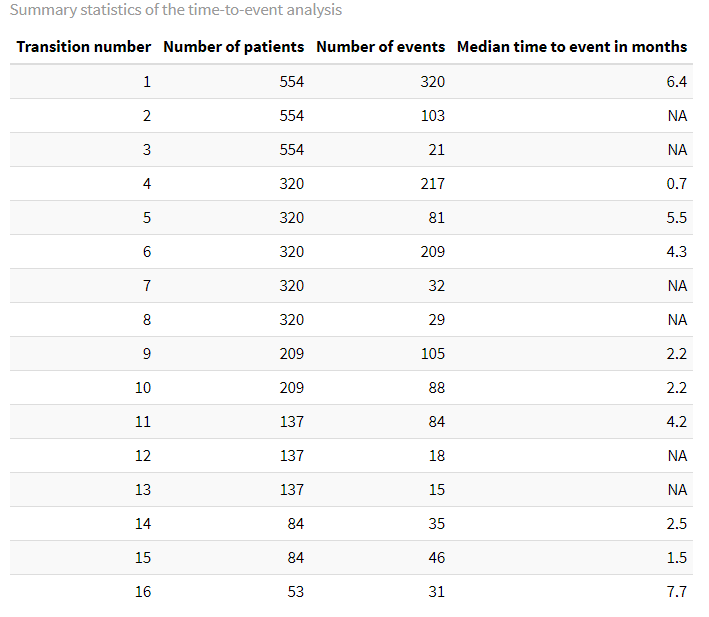


NA: Not available since median time to event was not reached

Note. Transition 1, PF1 -> PD1; Transition 2, PF1 -> PF2; Transition 3, PF1 -> D; Transition 4, PD1 -> PF2; Transition 5, PD1 -> D; Transition 6, PF2 -> PD2; Transition 7, PF2 -> PF3; Transition 8, PF2 -> D; Transition 9, PD2 -> PF3; Transition 10, PD2 -> D; Transition 11, PF3 -> PD3; Transition 12, PF3 -> PF4; Transition 13, PF3 -> D; Transition 14, PD3 -> PF4; Transition 15, PD3 -> D; Transition 16, PF4 -> D.

Table 3.5 AIC values per model per transition for BRAF-mutated melanoma patients with intermediate prognostic factors


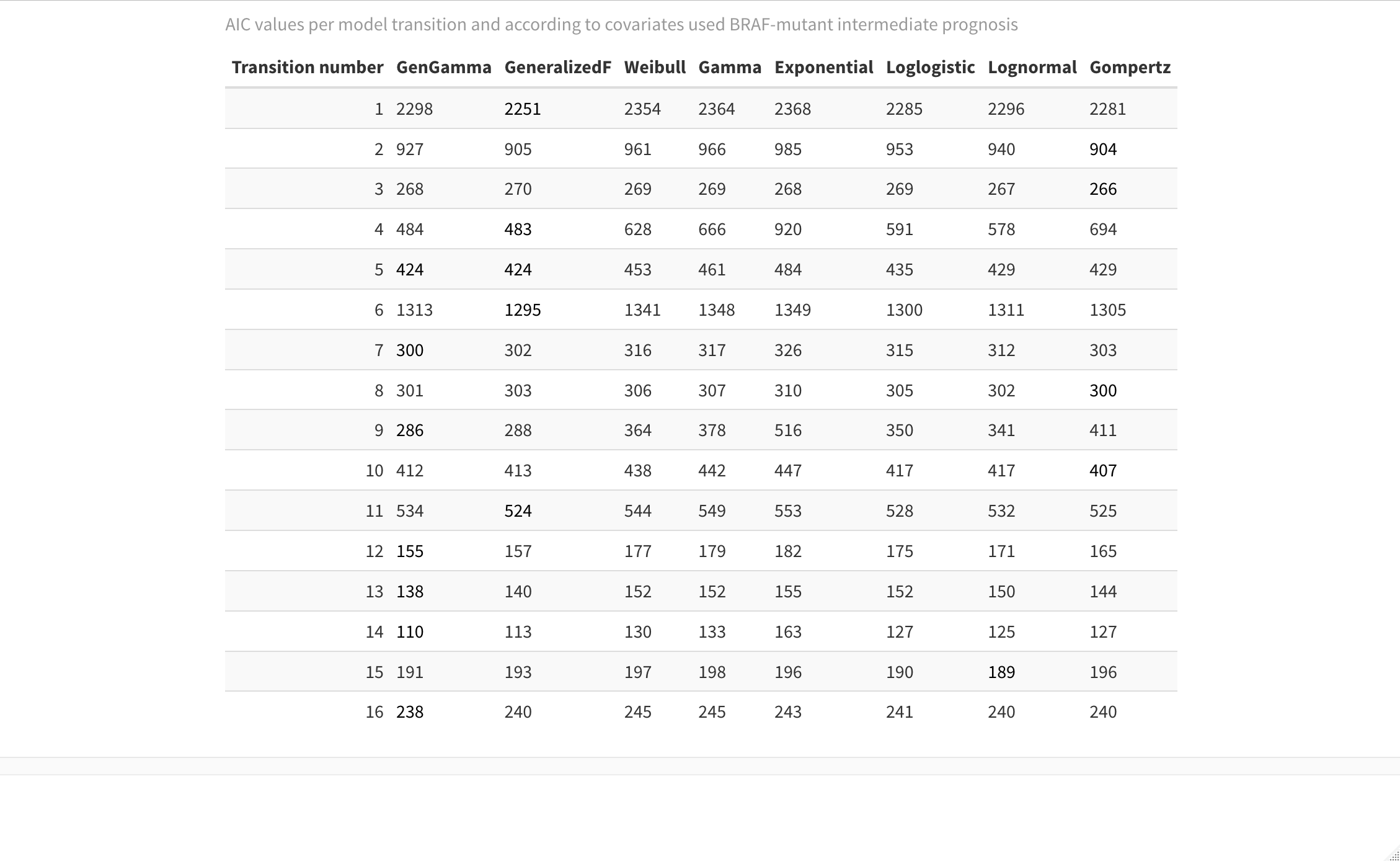


Table 3.6 BIC values per model per transition for BRAF-mutated melanoma patients with intermediate prognostic factors


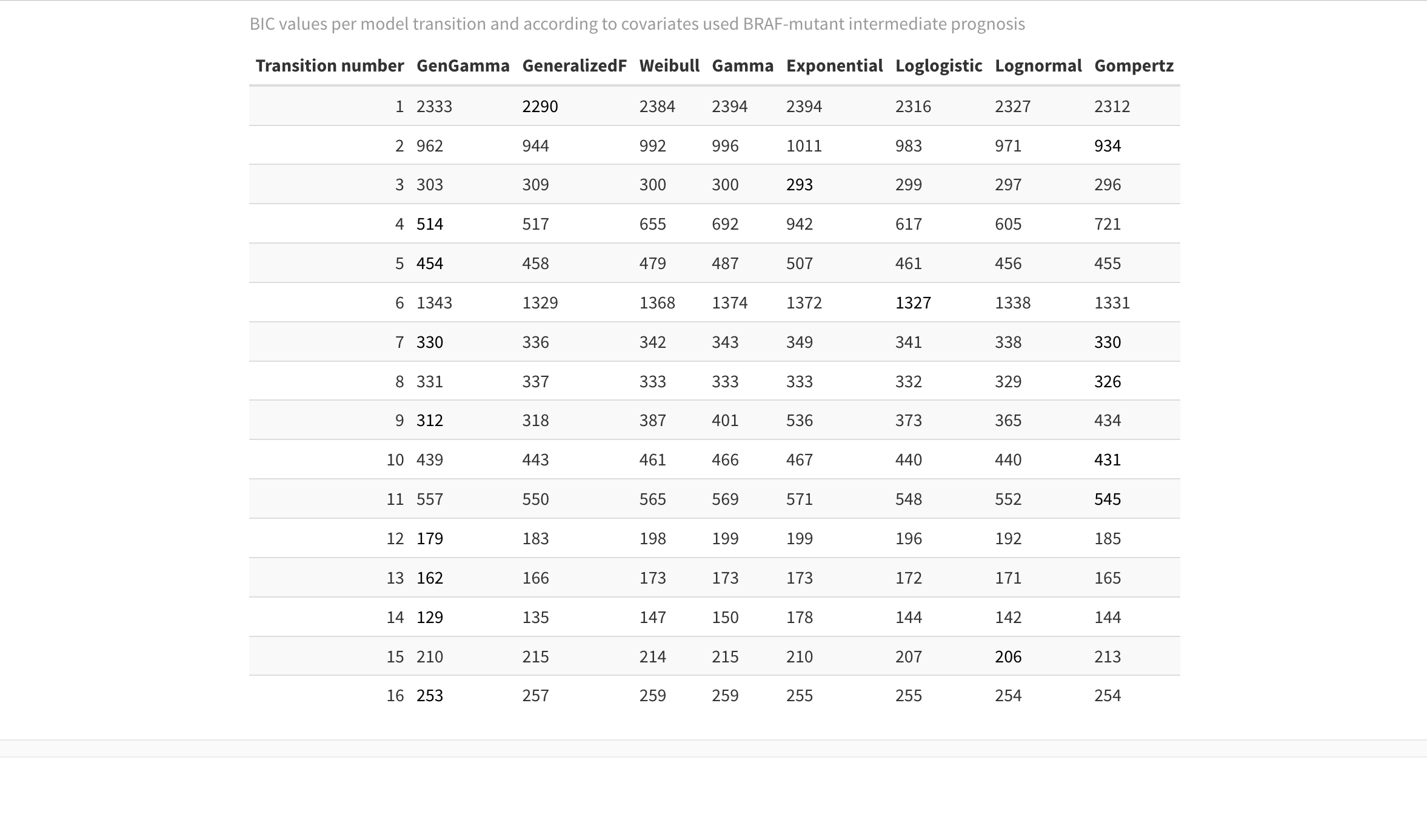


Figure 3.3 Overview of the fitted parametric curves for the transitions, time in months BRAF-mutant melanoma patients with intermediate prognostic factors


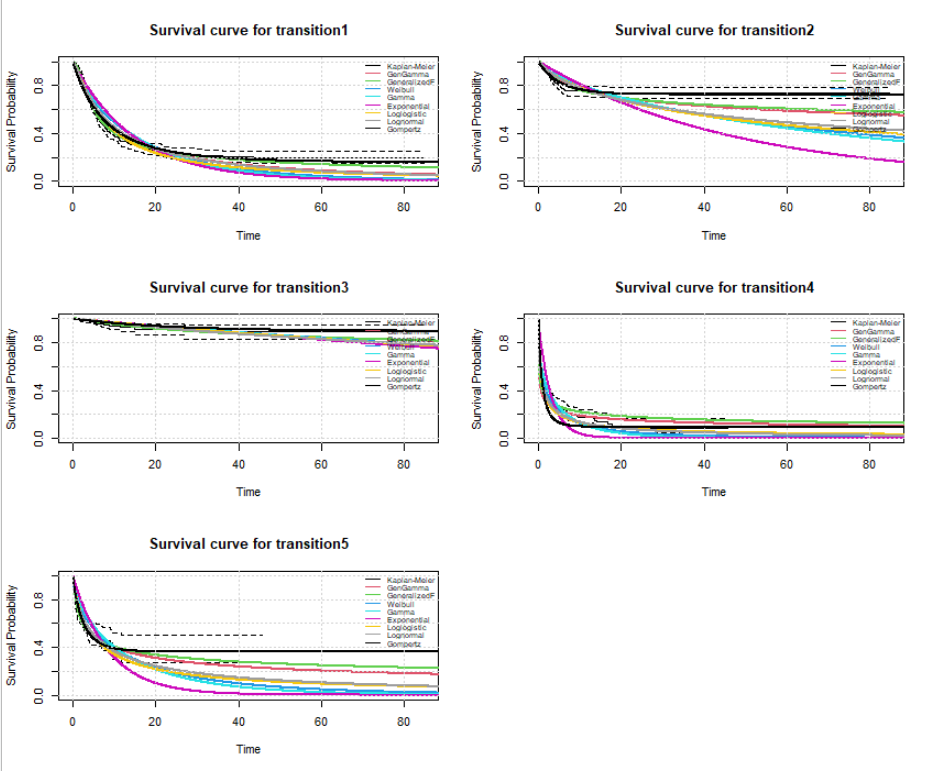


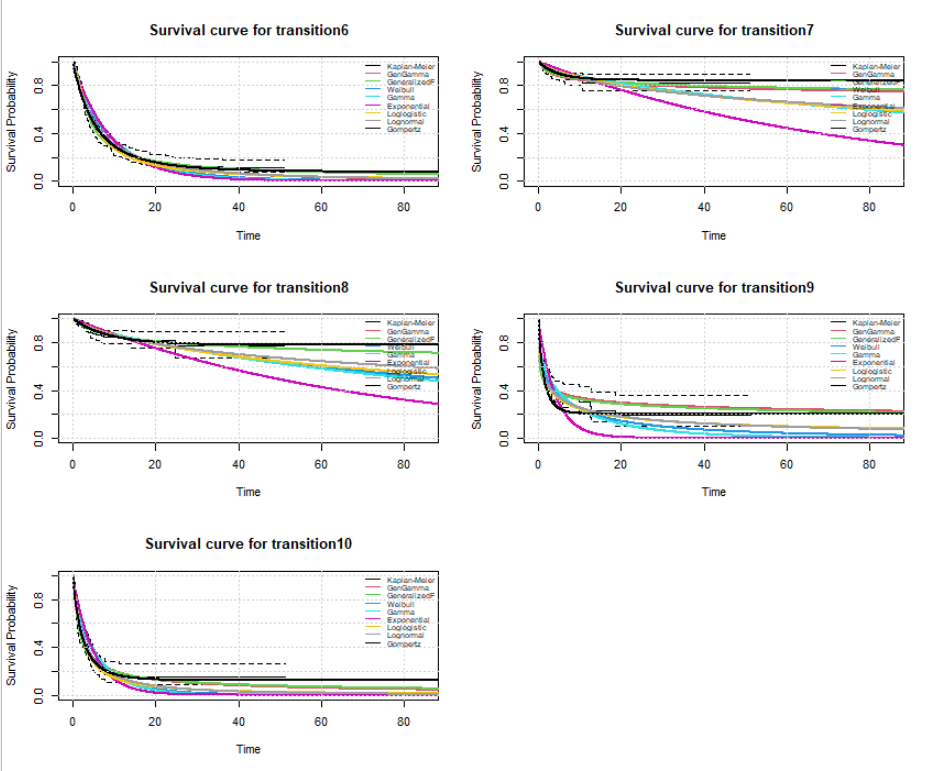


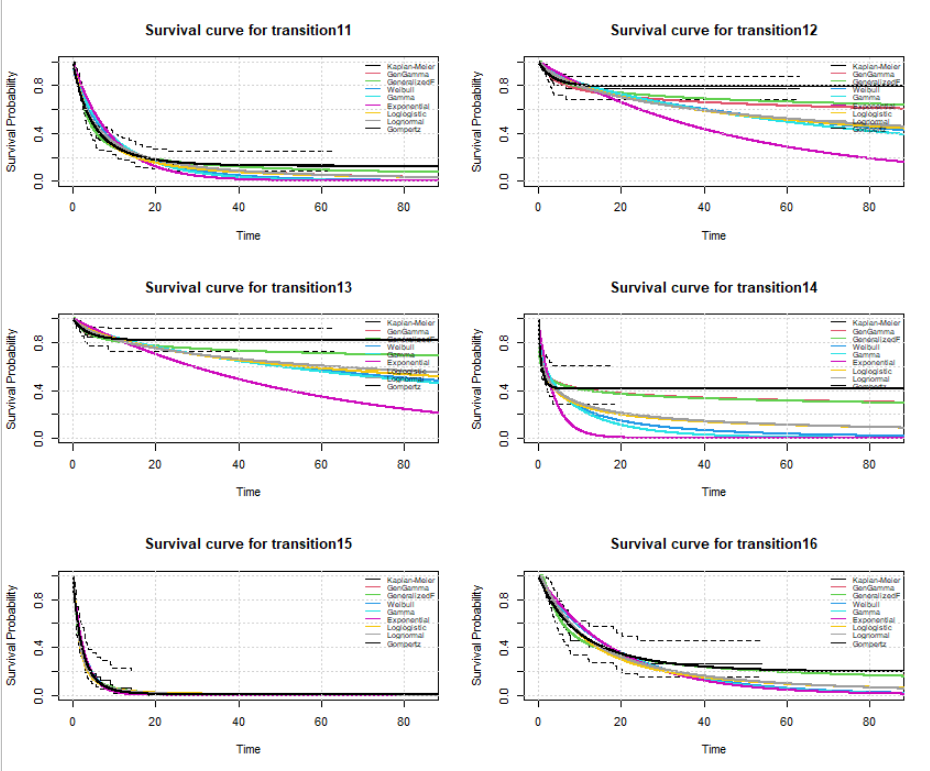


Figure 3.4 Overview of the selected parametric curves for the transitions, time in months BRAF-mutant melanoma patients with intermediate prognostic factors

Transition 1-5 BRAF-mutant Intermediate prognostic factors


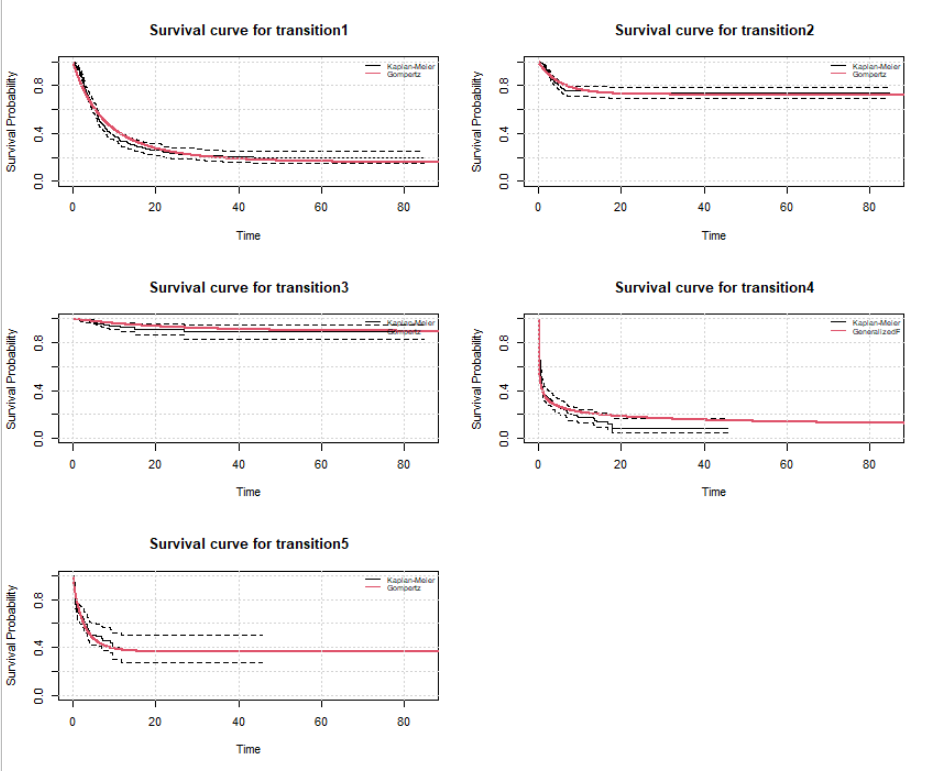


Transition 6-10 BRAF-mutant Intermediate prognostic factors


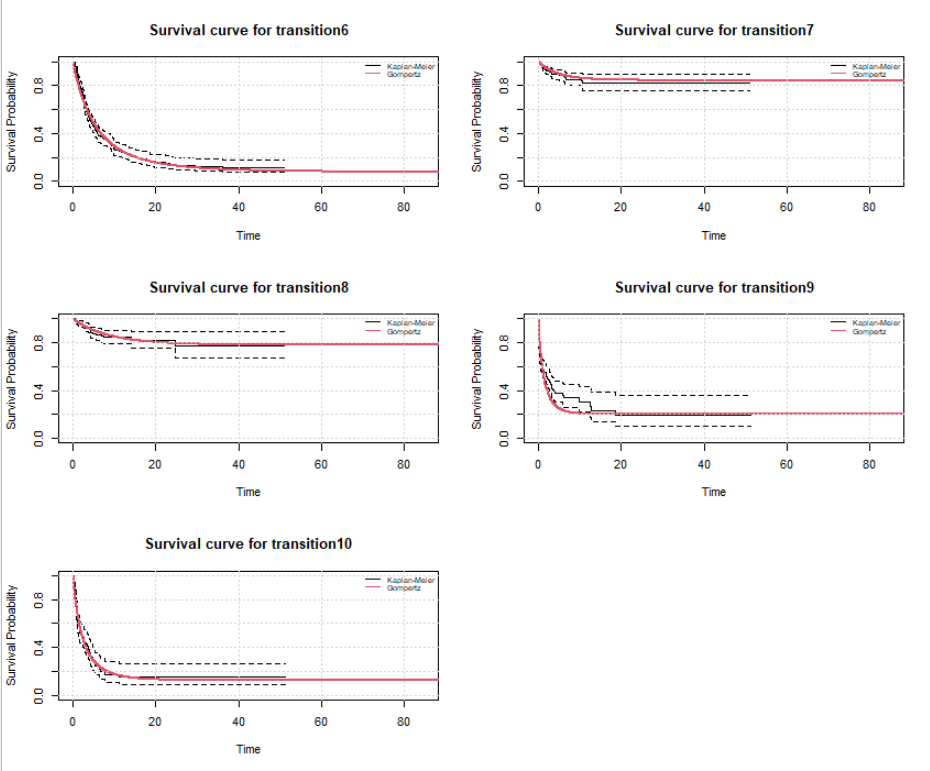


Transition 11-16 BRAF-mutant Intermediate prognostic factors


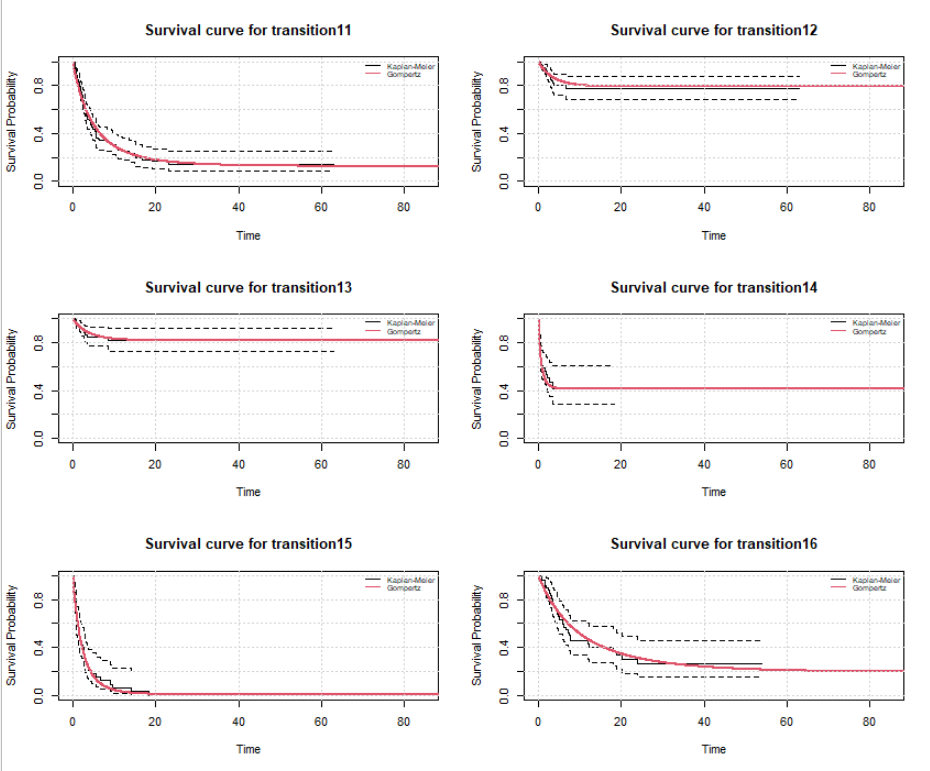


## BRAF wild-type melanoma favourable prognostic factors

In Table 3.7 below we present the summary statistics of the time to event analysis including number of patients included, number of events and median time to event. Table 3.8 present the AIC and Table 3.9 the BIC statistics. Figure 3.5 provides an overview of the fitted parametric distributions and Figure 3.6 an overview of the selected parametric distributions.

Table 3.7 Summary statistics of the time-to-event analysis BRAF wild-type melanoma patients with favourable prognostic factors


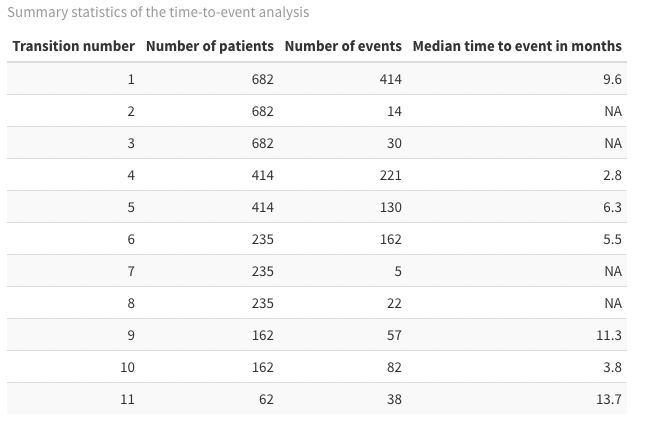


NA: Not available since median time to event was not reached

Note. Transition 1, PF1 -> PD1; Transition 2, PF1 -> PF2; Transition 3, PF1 -> D; Transition 4, PD1 -> PF2; Transition 5, PD1 -> D; Transition 6, PF2 -> PD2; Transition 7, PF2 -> PF3; Transition 8, PF2 -> D; Transition 9, PD2 -> PF3; Transition 10, PD2 -> D; Transition 11, PF3 -> D.

Table 3.8 AIC values per model per transition for BRAF wild-type melanoma patients with favourable prognostic factors


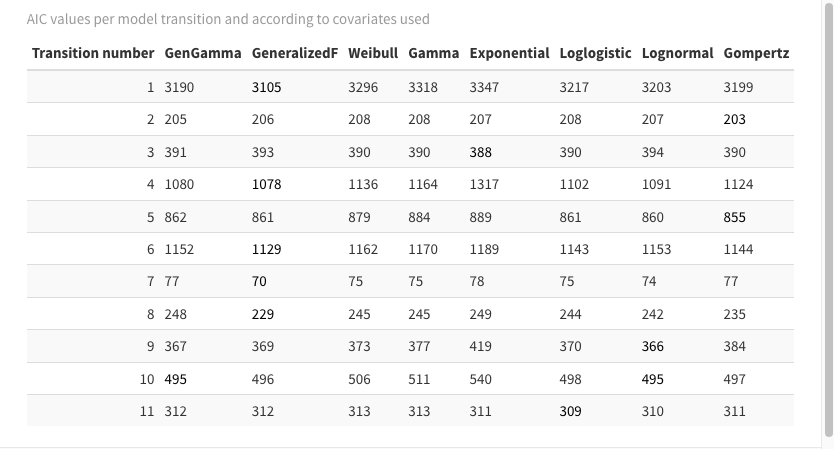


Table 3.9 BIC values per model per transition for BRAF wild-type melanoma patients with favourable prognostic factors


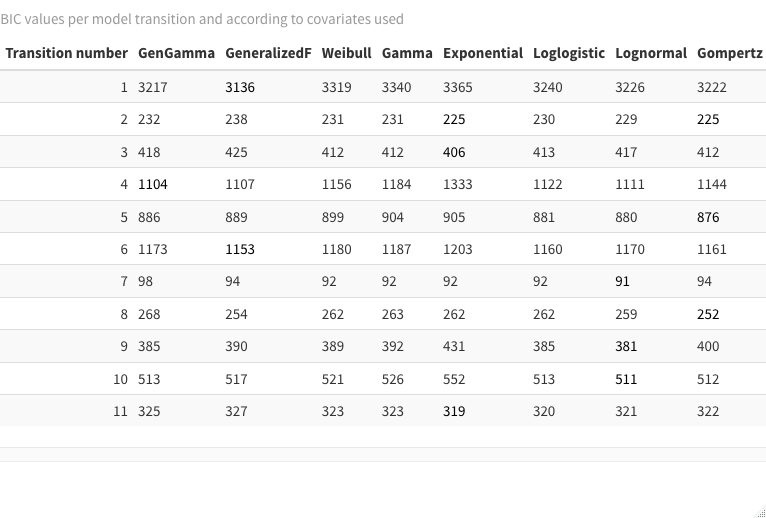


Figure 3.5 Overview of the fitted parametric curves for the transitions BRAF wild-type melanoma patients with favourable prognostic factors


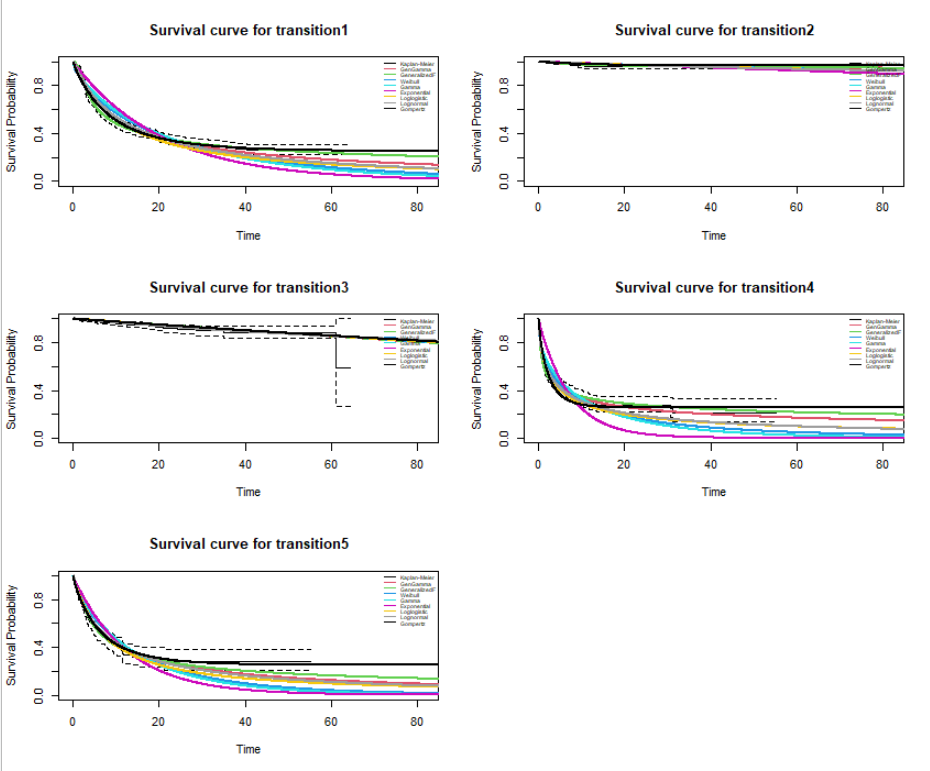


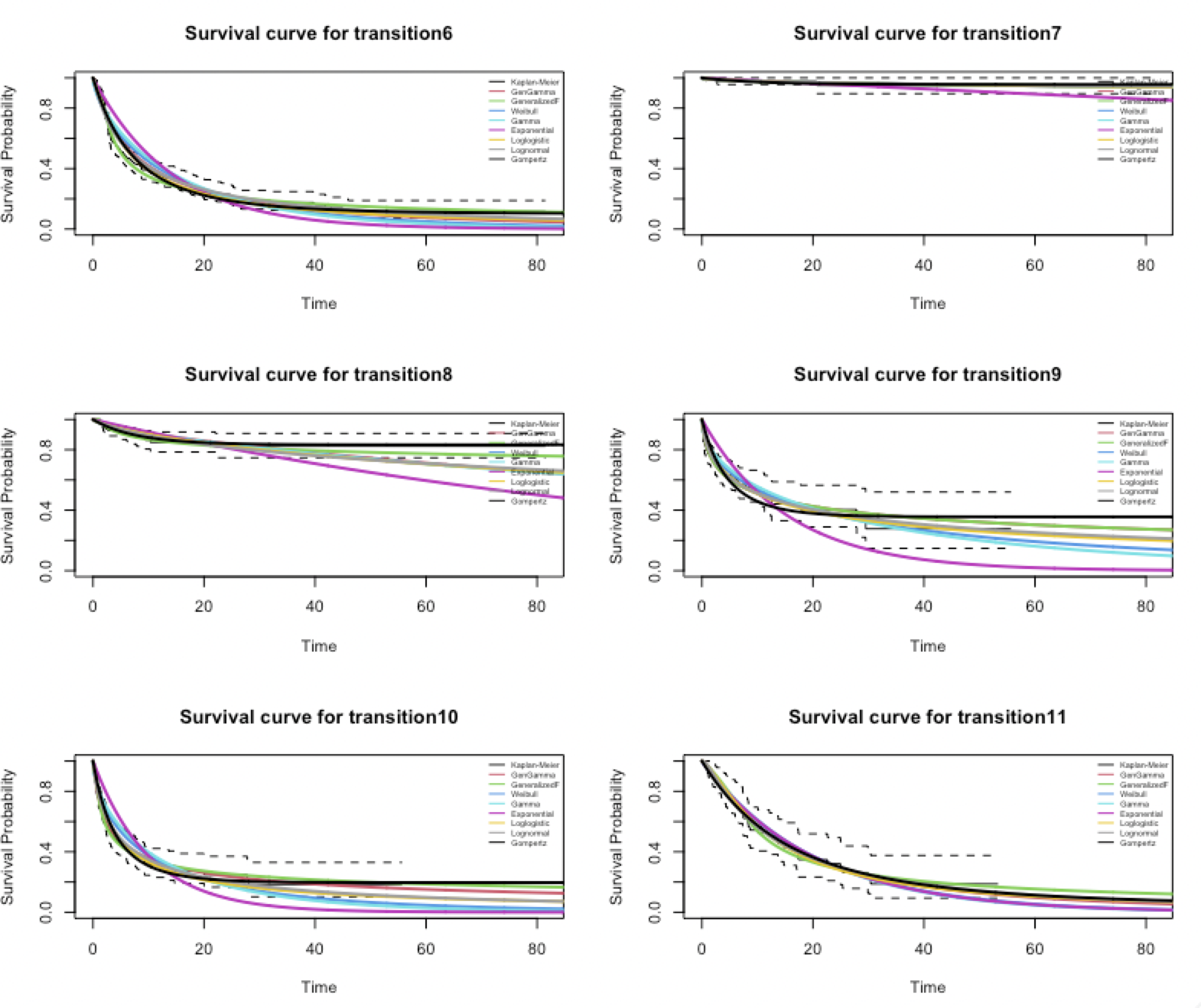


Figure 3.6 Overview of the selected parametric curves for the transitions BRAF wild-type melanoma patients with favourable prognostic factors


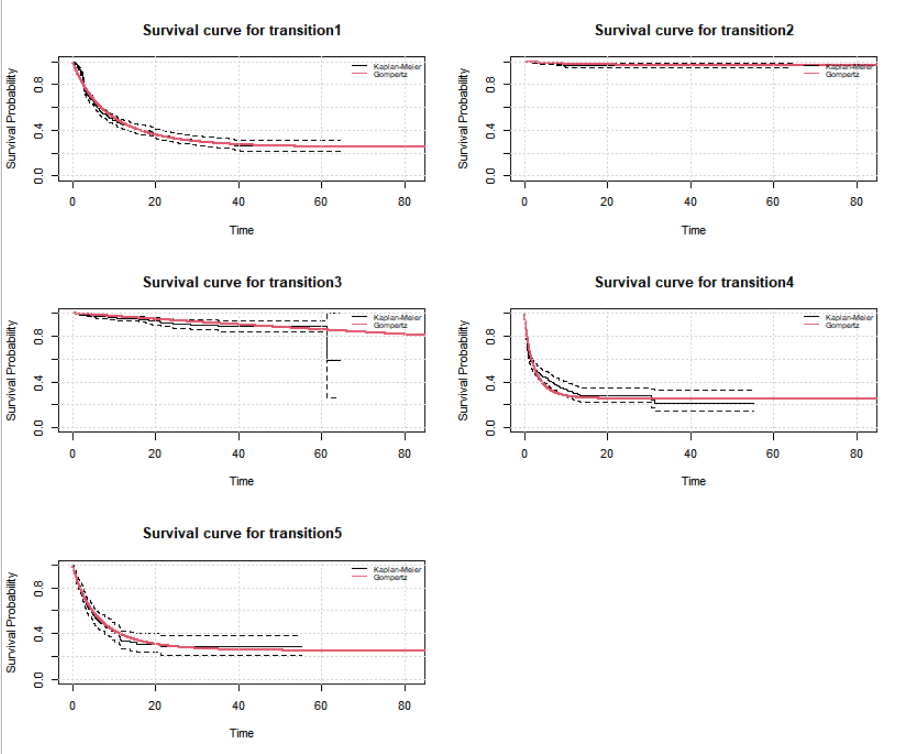


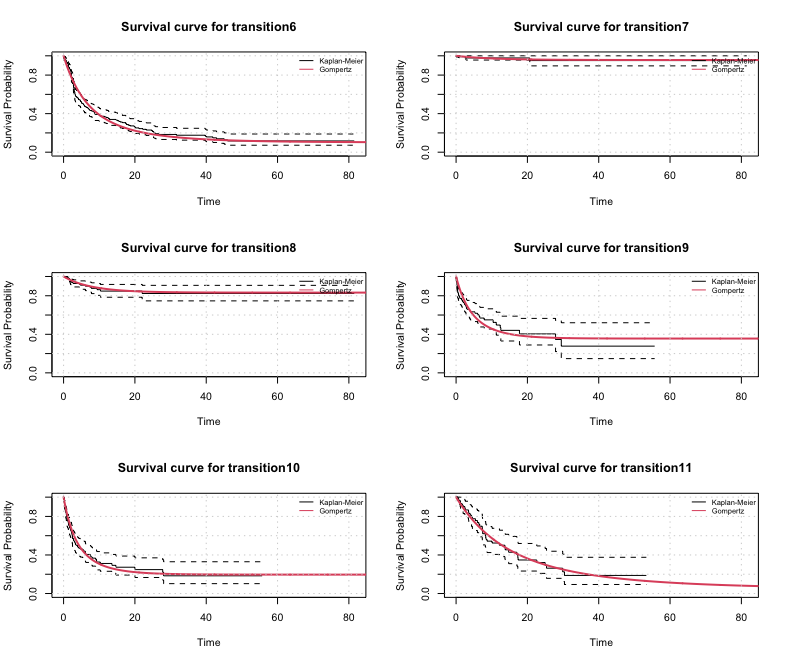


## BRAF wild-type melanoma intermediate prognostic factors

In Table 3.10 below we present the summary statistics of the time to event analysis including number of patients included, number of events and median time to event. Table 3.11 present the AIC and Table 3.12 the BIC statistics. Figure 3.7 provides an overview of the fitted parametric distributions and Figure 3.8 an overview of the selected parametric distributions.

Table 3.10 Summary statistics of the time-to-event analysis BRAF wild-type melanoma patients with intermediate prognostic factors


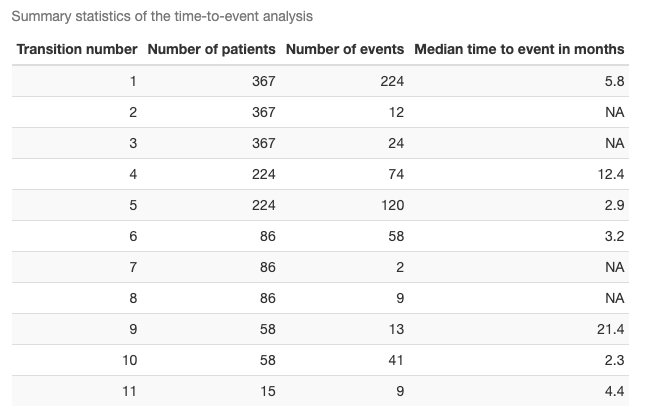


NA: Not available since median time to event was not reached

Note. Transition 1, PF1 -> PD1; Transition 2, PF1 -> PF2; Transition 3, PF1 -> D; Transition 4, PD1 -> PF2; Transition 5, PD1 -> D; Transition 6, PF2 -> PD2; Transition 7, PF2 -> PF3; Transition 8, PF2 -> D; Transition 9, PD2 -> PF3; Transition 10, PD2 -> D; Transition 11, PF3 -> D.

Table 3.11 AIC values per model per transition for BRAF wild-type melanoma patients with intermediate prognostic factors


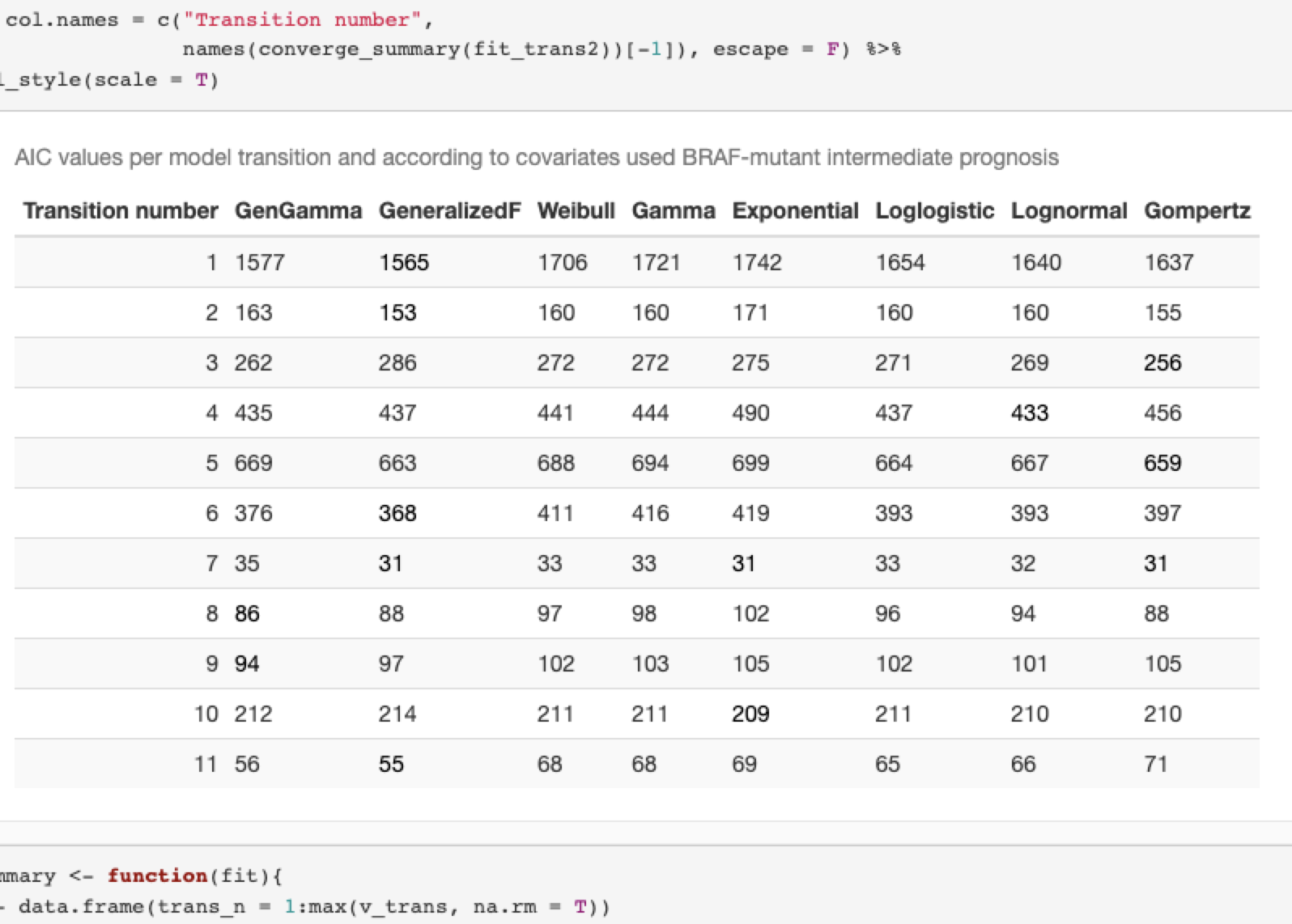


Table 3.12 BIC values per model per transition for BRAF wild-type melanoma patients with intermediate prognostic factors


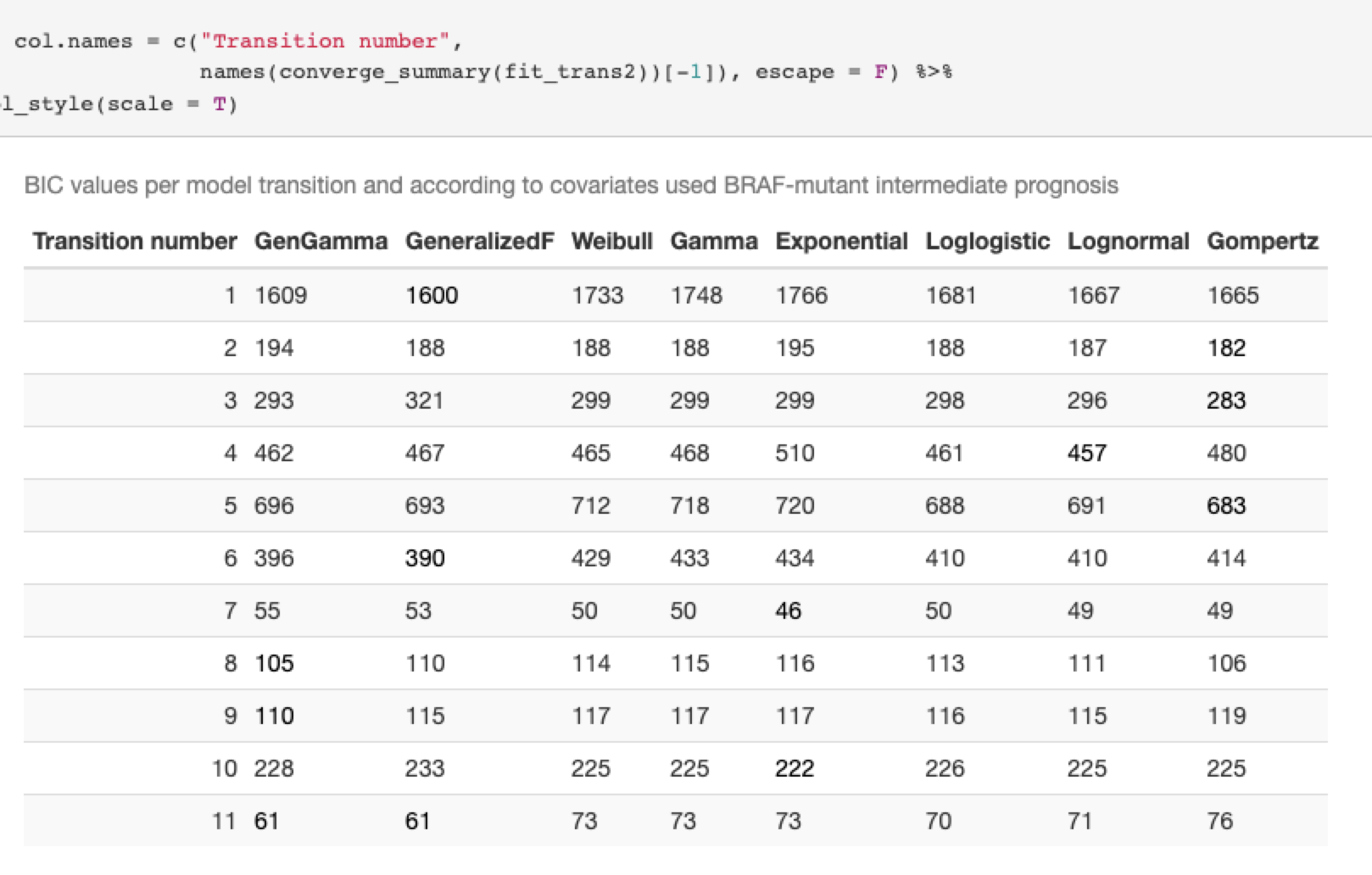


Figure 3.7 Overview of the fitted parametric curves for the transitions BRAF wild-type melanoma patients with intermediate prognostic factors


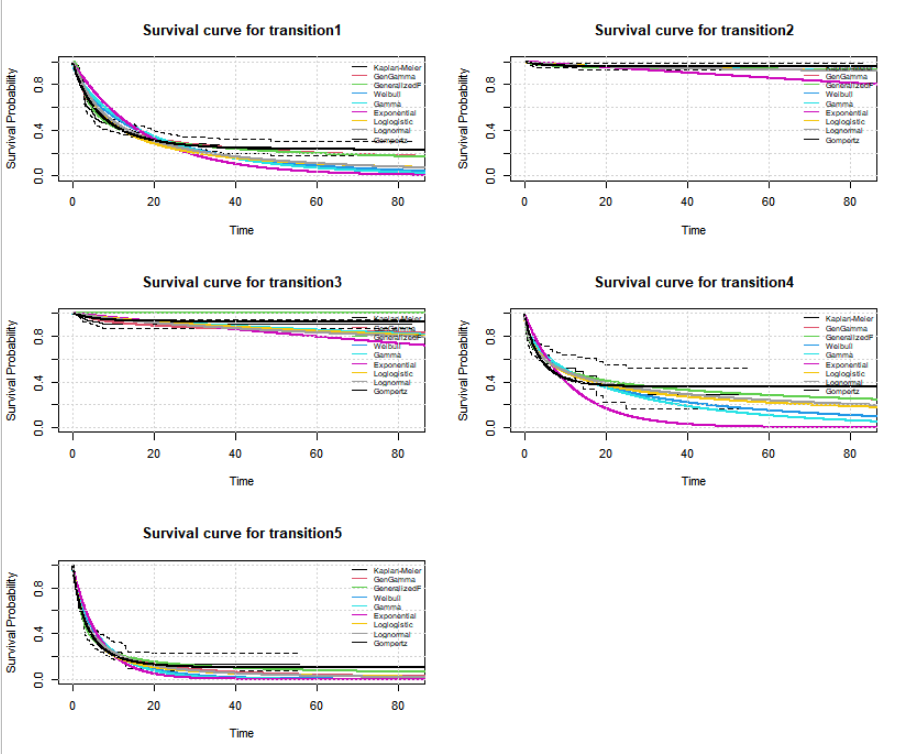


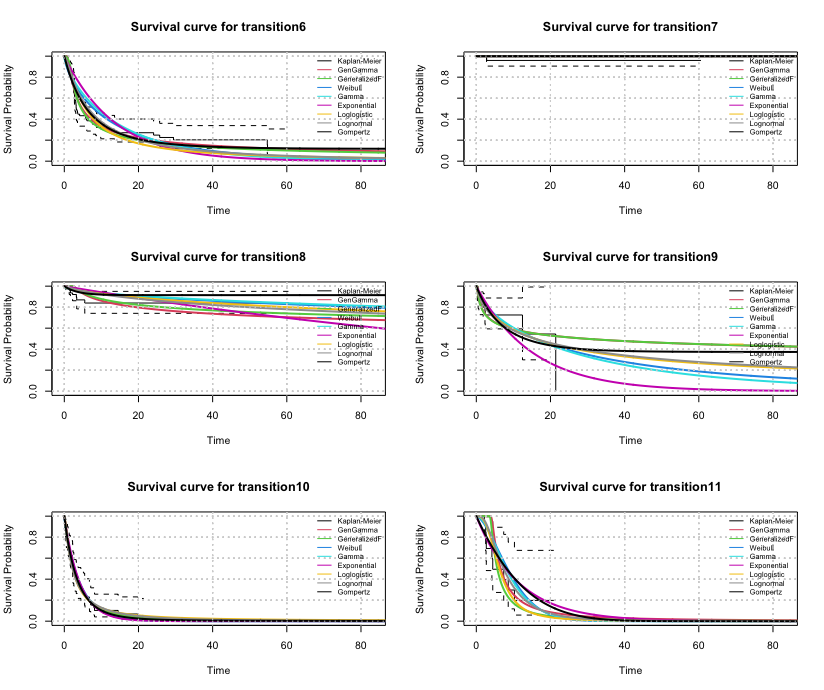


Figure 3.8 Overview of the selected parametric curves for the transitions BRAF wild-type melanoma patients with intermediate prognostic factors
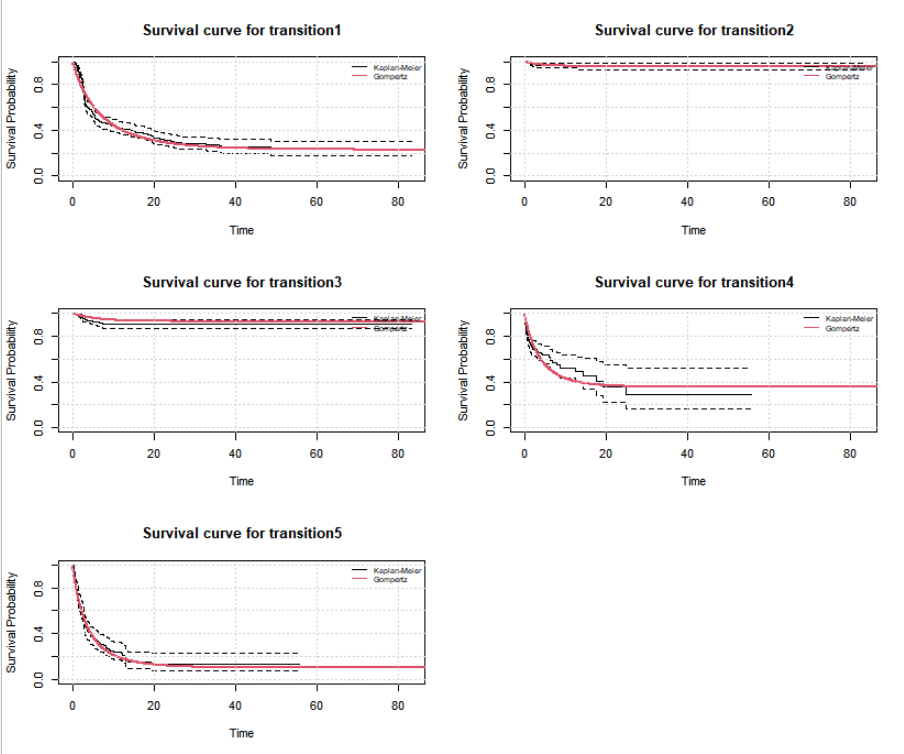


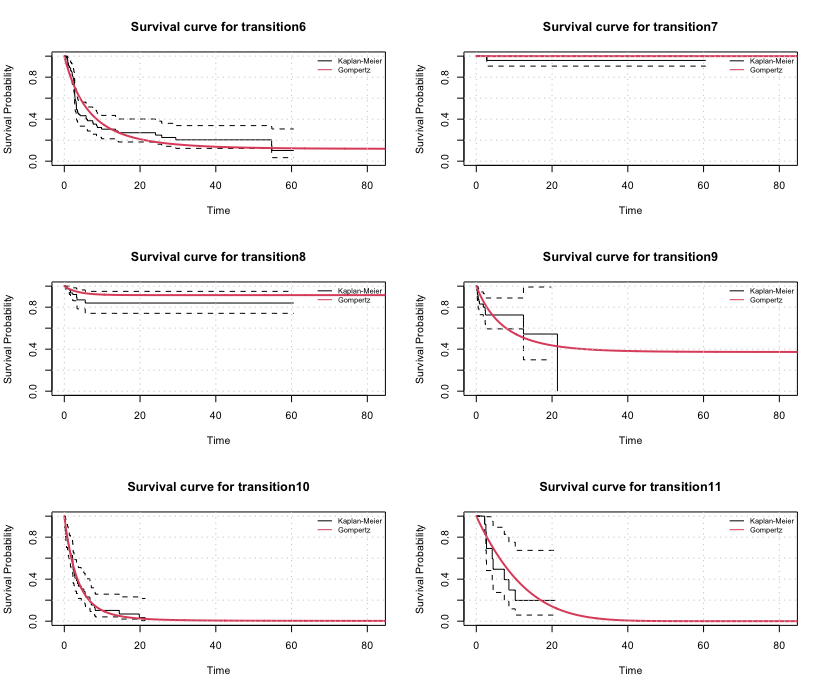


# Descriptives decision model

Table 4.1 Distributions for the parametric survival models and implementation of HRs from NMA

| Transition | Advanced melanoma with a BRAF mutation | | Advanced melanoma without a BRAF mutation | | Implementation of adjusted^b^ HRs from NMA - base case^c^ | Implementation of adjusted^b^ HRs from NMA - scenario 3 for sequence with immunotherapy in line 1^c^ | Implementation of adjusted^b^ HRs from NMA - scenario 3 for sequence with immunotherapy in line 2^c^ |
| --- | --- | --- | --- | --- | --- | --- | --- |
|  | Favourable prognosis | Intermediate prognosis | Favourable prognosis | Intermediate prognosis |  |  |  |
| 1: PF1 -> PD1 | Gompertz | Gompertz | Gompertz | Gompertz | HR1 PFS | HR1 PFS | HR1 PFS |
| 2: PF1 -> PF2 | Gompertz | Gompertz | Gompertz | Gompertz |  |  |  |
| 3: PF1 -> D | Gompertz | Gompertz | Gompertz | Gompertz | HR1 OS | HR1 OS | HR1 OS |
| 4: PD1 -> PF2 | Gompertz | Generalized F | Gompertz | Gompertz |  |  |  |
| 5: PD1 -> D | Gompertz | Gompertz | Gompertz | Gompertz | HR1 OS | HR1 OS | HR1 OS |
| 6: PF2 -> PD2 | Gompertz | Gompertz | Gompertz | Gompertz | HR2 PFS | HR2 PFS | HR2 PFS |
| 7: PF2 -> PF3 | Gompertz | Gompertz | Gompertz | Gompertz |  |  |  |
| 8: PF2 -> D | Gompertz | Gompertz | Gompertz | Gompertz | HR2 OS | HR1 OS | HR2 OS |
| 9: PD2 -> PF3 | Gompertz | Gompertz | Gompertz | Gompertz |  |  |  |
| 10: PD2 -> D | Gompertz | Gompertz | Gompertz | Gompertz | HR2 OS | HR1 OS | HR2 OS |
| 11: PF3 -> PD3^a^ | Gompertz | Gompertz | Gompertz | Gompertz | HR3 PFS | HR3 PFS | HR3 PFS |
| 12: PF3 -> PF4 | Gompertz | Gompertz |  |  |  |  |  |
| 13: PF3 -> D | Gompertz | Gompertz |  |  | HR3 OS | HR1 OS | HR2 OS |
| 14: PD3 -> PF4 | Gompertz | Gompertz |  |  |  |  |  |
| 15: PD3 -> D | Gompertz | Gompertz |  |  | HR3 OS | HR1 OS | HR2 OS |
| 16: PF4 -> D | Gompertz | Gompertz |  |  |  |  |  |

^a^ In the model for advanced melanoma without a BRAF mutation this transition represents PF3->D.

^b^ The HRs are adjusted with equation 1, except for the first treatment sequence, i.e. mix (observed in clinical practice).

^c^ HR1 refers to the hazard ratio for either PFS or OS related to the first-line treatment within the sequence that is evaluated; HR2 refers to the hazard ratio for either PFS or OS related to the second-line treatment within the sequence that is evaluated; HR3 refers to the hazard ratio for either PFS or OS related to the third-line treatment within the sequence that is evaluated.

Abbreviations: PF1, progression-free during or after treatment line 1; PD1, progressive disease after treatment line 1; PF2, progression-free during or after treatment line 2; PD2, progressive diseases after treatment line 2; PF3, progression-free during or after treatment line 3; PD3, progressive disease after treatment line 3; PF4, progression-free during and progressive disease after subsequent therapies; D, death.

# Overview of modelled treatment sequences

Table 5.1 Modelled treatment sequences for patients with a BRAF mutation with favourable and intermediate prognostic factors

| **First line treatment** | **Second line treatment** | **Third line treatment** |
| --- | --- | --- |
| mix (observed in clinical practice) | mix (observed in clinical practice) | mix (observed in clinical practice) |
| chemotherapy | chemotherapy | Chemotherapy |
| dabrafenib plus trametinib | nivolumab | real-world treatment mix^a^ |
| dabrafenib plus trametinib | pembrolizumab | real-world treatment mix^a^ |
| dabrafenib plus trametinib | ipilimumab plus nivolumab | real-world treatment mix^a^ |
| vemurafenib plus cobimetinib | nivolumab | real-world treatment mix^a^ |
| vemurafenib plus cobimetinib | pembrolizumab | real-world treatment mix^a^ |
| vemurafenib plus cobimetinib | ipilimumab plus nivolumab | real-world treatment mix^a^ |
| encorafenib plus binimetinib | nivolumab | real-world treatment mix^a^ |
| encorafenib plus binimetinib | pembrolizumab | real-world treatment mix^a^ |
| encorafenib plus binimetinib | ipilimumab plus nivolumab | real-world treatment mix^a^ |
| nivolumab | ipilimumab | real-world treatment mix^a^ |
| nivolumab | dabrafenib plus trametinib | real-world treatment mix^a^ |
| nivolumab | vemurafenib plus cobimetinib | real-world treatment mix^a^ |
| nivolumab | encorafenib plus binimetinib | real-world treatment mix^a^ |
| nivolumab | ipilimumab plus nivolumab | real-world treatment mix^a^ |
| pembrolizumab | ipilimumab | real-world treatment mix^a^ |
| pembrolizumab | dabrafenib plus trametinib | real-world treatment mix^a^ |
| pembrolizumab | vemurafenib plus cobimetinib | real-world treatment mix^a^ |
| pembrolizumab | encorafenib plus binimetinib | real-world treatment mix^a^ |
| nivolumab plus ipilimumab | dabrafenib plus trametinib | real-world treatment mix^a^ |
| nivolumab plus ipilimumab | vemurafenib plus cobimetinib | real-world treatment mix^a^ |
| nivolumab plus ipilimumab | encorafenib plus binimetinib | real-world treatment mix^a^ |

^a^ Real-world treatment mix refers to the total mix of third-line therapies observed in clinical practice (regardless of the type of first- and second-line treatment).

Table 5.2 Modelled treatment sequences for patients without a BRAF mutation with favourable and intermediate prognostic factors

| **First line treatment** | **Second line treatment** |
| --- | --- |
| mix (observed in clinical practice) | mix (observed in clinical practice) |
| chemotherapy | chemotherapy |
| nivolumab | real-world treatment mix^a^ |
| nivolumab | ipilimumab |
| nivolumab | ipilimumab plus nivolumab |
| pembrolizumab | real-world treatment mix^a^ |
| pembrolizumab | ipilimumab |
| pembrolizumab | ipilimumab plus nivolumab |
| nivolumab plus ipilimumab | real-world treatment mix^a^ |

^a^ Real-world treatment mix refers to the total mix of second-line therapies observed in clinical practice (regardless of the type of first-line treatment).

# Outcomes decision model patients without a BRAF mutation

Table 6.1 Life expectancy (in years) in the base case and different scenarios of patients without a BRAF mutation stratified by prognostic factors

| First line treatment | Mix (observed in clinical practice)^b^ | Chemotherapy | nivolumab | | | pembrolizumab | | | nivolumab plus ipilimumab |
| --- | --- | --- | --- | --- | --- | --- | --- | --- | --- |
| Second line treatment | Mix (observed in clinical practice)^b^ | Chemotherapy | real-world treatment mix^a^ | ipilimumab | nivolumab plus ipilimumab | real-world treatment mix^a^ | ipilimumab | nivolumab plus ipilimumab | real-world treatment mix^a^ |
| Patients with FAVOURABLE prognostic factors | | | | | | | | | |
| Base case | 7.1 (5.1 - 9.1) | 3 (2.2 - 3.7) | 8.6 (5.7 - 11.5) | 8.2 (5.2 - 11.3) | 9.9 (6.7 - 13.1) | 8.2 (5.1 - 11.3) | 7.8 (4.5 - 11.1) | 9.5 (6.1 - 12.9) | 10.1 (6.4 - 13.9) |
| Scenario 1) | Not applicable | | | | | | | | |
| Scenario 2) | Not applicable | | | | | | | | |
| Scenario 3) | 7.1 (5.1 - 9.1) | 3 (2.2 - 3.7) | 8.9 (5.7 - 12.1) | 8.7 (5.4 - 11.9) | 10.2 (6.8 - 13.6) | 8.4 (4.6 - 12.1) | 8.1 (4.3 - 11.9) | 9.8 (6.1 - 13.4) | 10.8 (6.6 - 14.9) |
| Scenario 4) | 4.3 (3.8 - 4.8) | 2.2 (1.8 - 2.5) | 5 (4.3 - 5.6) | 4.8 (4 - 5.5) | 5.5 (4.7 - 6.3) | 4.8 (3.9 - 5.7) | 4.6 (3.5 - 5.6) | 5.3 (4.3 - 6.4) | 5.5 (4.7 - 6.3) |
| Patients with INTERMEDIATE prognostic factors | | | | | | | | | |
| Base case | 5.6 (4.6 - 6.7) | 1.6 (1.3 - 2) | 6.9 (5.1 - 8.8) | 6.6 (4.7 - 8.6) | 7.8 (5.7 - 10) | 6.4 (4 - 8.8) | 6.1 (3.6 - 8.6) | 7.3 (4.6 - 10.1) | 8.7 (6.2 - 11.2) |
| Scenario 1) | Not applicable | | | | | | | | |
| Scenario 2) | Not applicable | | | | | | | | |
| Scenario 3) | 5.6 (4.6 - 6.7) | 1.6 (1.3 - 2) | 7 (5 - 9) | 6.7 (4.7 - 8.8) | 7.9 (5.7 - 10.1) | 6.5 (3.9 - 9.1) | 6.2 (3.6 - 8.9) | 7.5 (4.6 - 10.3) | 8.9 (6.3 - 11.6) |
| Scenario 4) | 3.3 (2.8 - 3.8) | 1.2 (1 - 1.5) | 3.8 (3 - 4.6) | 3.7 (2.8 - 4.5) | 4.2 (3.3 - 5.1) | 3.6 (2.6 - 4.6) | 3.4 (2.3 - 4.5) | 4 (2.8 - 5.1) | 4.5 (3.6 - 5.5) |

^a^ Real-world treatment mix refers to the total mix of second-line therapies observed in clinical practice (regardless of the type of first-line treatment).

^b^ Outcomes of current treatment (mix [observed in clinical practice]) did not require an adjustment of the HRs with equation 1.

Note. The life expectancy is derived from the probabilistic sensitivity analyses.
